# Electroacupuncture alleviates migraine through CXCL13/CXCR5-mediated communication

**DOI:** 10.1186/s13020-026-01338-8

**Published:** 2026-02-02

**Authors:** Yine Song, Shaoru Zhao, Peiyue Peng, Yuhan Liu, Chengcheng Zhang, Baicheng Cao, Yuxi Luo, Xin Yang, Jiangyan Wei, Xiaobo Ge, Luopeng Zhao, Bin Li, Lu Liu

**Affiliations:** Department of Acupuncture and Moxibustion, Beijing Hospital of Traditional Chinese Medicine, Beijing Key Laboratory of Acupuncture Neuromodulation, Capital Medical University, No. 23 Meishuguan Houjie, Beijing, 100010 China

**Keywords:** Migraine, Neuroinflammation, CXCR5, Neuron, Cross-talk, FOXO3

## Abstract

**Rationale:**

Chronic migraine is characterized by persistent trigeminal sensitization and neuroinflammation. however, the molecular mechanisms underlying its maintenance and mediate the therapeutic effects of acupuncture remian incompletely understood.

**Method:**

A chronic migraine–like state was induced in mice by repeated dural inflammatory soup (IS), followed by electroacupuncture (EA). Behavioral hypersensitivity was assessed, and molecular changes in the spinal trigeminal nucleus caudalis (Sp5C) were analyzed using transcriptomic, biochemical, and functional approaches.

**Result:**

Repeated inflammatory stimulation markedly increased CXCL13 and CXCR5 expression and ERK phosphorylation in the Sp5C, accompanied by mechanical allodynia, thermal hyperalgesia, glial activation, and elevated IL-6 and CCL2 levels. EA significantly attenuated pain hypersensitivity and reduced CXCL13/CXCR5 expression, ERK activation, glial reactivity, and inflammatory mediator release. EA also decreased migraine-related neuropeptides and synaptic plasticity markers, including substance P, PACAP, and NR2B. Functional manipulation experiments demonstrated bidirectional regulation of pain behaviors by CXCR5, establishing its causal role in chronic migraine–like sensitization. MicroRNA profiling identified a dysregulated miRNA signature converging on the transcription factor FOXO3, which indirectly regulated CXCR5 transcription, defining a miRNA–FOXO3–CXCR5 regulatory pathway.

**Conclusion:**

Our study reveals the CXCL13/CXCR5/ERK axis as a previously unrecognized pathway in migraine neuroinflammation and demonstrates electroacupuncture's multimodal therapeutic mechanisms. These findings provide: (1) novel mechanistic insights into migraine pathophysiology through CXCR5-mediated signaling, and (2) translational implications for chronic migraine treatment by targeting the CXCL13/CXCR5/ERK axis. This work establishes a foundation for future development of targeted therapies and validates electroacupuncture as a viable intervention for migraine management.

**Supplementary Information:**

The online version contains supplementary material available at 10.1186/s13020-026-01338-8.

## Background

Migraine has emerged as a leading global health challenge, with prevalence surging by 58.2% over the past three decades to affect approximately 1.16 billion people worldwide, making it the third highest cause of disability-adjusted life years [1–3]. Current therapeutic strategies are bifurcated into acute abortive treatments and preventive approaches. While triptans and NSAIDs remain first-line options for acute attacks, and β-blockers/topiramate serve as conventional preventatives [4–6], the landscape has been transformed by CGRP-targeted biologics—monoclonal antibodies demonstrating > 50% responder rates in clinical trials [7]. Despite these advances, significant treatment gaps persist: novel gepants/ditans face cost and accessibility barriers [8], while traditional medications carry risks of medication-overuse headache and diminishing efficacy over time [5]. These limitations are particularly pronounced in refractory cases, creating an urgent need for alternative interventions with improved safety profiles and sustainable efficacy.

Emerging evidence underscores neuroinflammation as a central driver of migraine pathogenesis. Preclinical studies utilizing trigeminovascular system (TGVS) models have established that neuroinflammatory mechanisms mediate migraine-related pain sensitization through heightened neuronal excitability and synaptic potentiation in key regions like the Sp5C [9, 10]. This process involves a self-perpetuating cycle where activated neurons release neuropeptides that stimulate glial cells and immune mediators, culminating in amplified inflammatory signaling and sustained central sensitization [11, 12].

The CXCL13/CXCR5 chemokine axis has emerged as a critical regulator of this neuroinflammatory cascade. As a potent chemoattractant, CXCL13 binds specifically to CXCR5 to orchestrate neuroimmune interactions that maintain pain hypersensitivity [13, 14]. Notably, CXCL13 upregulation in the spinal cord drives both pro-inflammatory cytokine release and astrocyte activation, mechanisms directly implicated in neuropathic pain pathogenesis [15–17]. Recent work demonstrates that astrocyte-derived CXCL13 drives ERK-mediated neuroinflammation, while targeted disruption of CXCL13/CXCR5 signaling attenuates pain behaviors by suppressing ERK phosphorylation and glial activation [18]. These findings position the CXCL13/CXCR5/ERK pathway as a promising therapeutic target for migraine-related central sensitization.

Acupuncture has demonstrated clinically significant analgesic effects in migraine treatment, with multiple randomized controlled trials confirming its ability to reduce attack frequency and pain intensity [19–21]. Our preclinical studies further substantiate these findings, revealing that electroacupuncture (EA) effectively modulates migraine-associated neuroinflammation [22–24]. MicroRNA sequencing enables precise identification of functionally relevant target genes, uncovering key regulatory mechanisms in disease pathways. Using the established inflammatory soup dural injection model [25], our results revealed that EA mediates its analgesic effects by coordinately downregulating the CXCL13/CXCR5 chemokine axis, suppressing ERK signaling, and reducing key inflammatory mediators, including CCL2 and IL-6. This multimodal action extends to the regulation of pain-related neuropeptides and excitatory receptors, suggesting acupuncture's unique capacity to simultaneously target both peripheral trigeminovascular activation and central sensitization mechanisms. MicroRNA sequencing identified *Foxo3* as an intermediary gene, and subsequent validation showed that acupuncture also modulates FOXO3 protein expression, supporting a potential microRNA–FOXO3–CXCR5 transcriptional regulatory relationship. Together, our findings provide insight into how acupuncture may modulate the interconnected processes of neuroinflammation and neuronal hyperexcitability underlying migraine.

## Materials and methods

### Animals

Consistent with established migraine research protocols [26–28], adult male C57BL/6J mice (6-week-old, 18–21 g) were obtained from the National Institutes for Food and Drug Control (Beijing, China) to standardize experimental conditions by eliminating potential hormonal variability. Animals were housed in specific pathogen-free conditions under controlled temperature (22 ± 1 °C) and humidity (50 ± 10%) with a 12-h light/dark cycle, receiving ad libitum access to food and water. Following a minimum 7-day acclimation period to the facility environment, mice were progressively habituated to handling and experimental apparatuses prior to testing. All experimental protocols were approved by the Beijing Animal Experimental Institution Review Committee (BJTCM-R-2021–11-03) and strictly followed the ethical guidelines of the International Association for the Study of Pain for nociception research. The work has been reported in accordance with the ARRIVE guidelines (Animals in Research: Reporting In Vivo Experiments) [29]. Researchers received specialized training in rodent pain assessment to ensure optimal animal welfare during behavioral tests and postoperative care.

### Establishment of the mice model of migraine-like pain

The study employed the well-validated dural IS injection model to replicate key pathophysiological features of human migraine, including trigeminovascular activation and neuroinflammatory responses as established in prior research [30]. Under sodium pentobarbital anesthesia, a 1 mm cranial window was carefully created using a pre-cooled dental drill (DH-0 Pin Vise, Plastics One) at the stereotaxically defined location (Fig. S1A), exposing the intact dura mater. Model animals received four dural infusions (days 1, 3, 5, and 7) of 5 μL IS containing 1 mM bradykinin, 1 mM serotonin, 1 mM histamine, and 0.1 mM prostaglandin E2 in PBS [31], while controls received equivalent volumes of PBS. Throughout the procedure, core temperature was maintained at 38 °C using a feedback-regulated heating pad, with continuous monitoring of respiratory rate, cardiac function, and peripheral perfusion. Animals showing neurological impairment or distress signs were humanely excluded per predefined criteria. All IS challenges were performed three weeks after adenoviral vector administration to ensure stable transgene expression.

### Adenovirus vectors production and intra-Sp5C injection

To investigate the functional role of CXCR5 in migraine pathophysiology, we performed stereotaxic delivery of adeno-associated viral (AAV) vectors for targeted manipulation of *Cxcr5* expression in the Sp5C region. All viral constructs were designed and commercially produced by Shanghai GeneChem. The experimental vectors included AAV-GV388 vectors (3.14 × 10^12^ viral genomes/mL) for *Cxcr5* overexpression along with matched negative controls, and AAV-GV478 vectors encoding either *Cxcr5*-targeting shRNA (5'-CCA TCA CCT TGT GTG AAT T-3') or non-targeting control shRNA (5'-TTC TCC GAA CGT GTC ACGT-3').

For stereotaxic surgery, mice were anesthetized with isoflurane and securely positioned in a digital stereotaxic apparatus. Using anatomical landmarks referenced to bregma, we determined the coordinates for Sp5C to be –8.03 mm posterior, ± 1.55 mm lateral, and 4.05 mm ventral to the skull surface, based on Paxinos and Franklin’s The Mouse Brain in Stereotaxic Coordinates (Fig. S2A-B). Viral suspensions were carefully infused into the Sp5C through glass micropipettes connected to a microprocessor-controlled injection system, delivering 0.5 μL per side at a precisely controlled flow rate of 0.02 μL/min. To ensure optimal viral distribution and minimize reflux, the injection pipettes remained in place for at least five minutes following each infusion before being slowly withdrawn. We maintained strict aseptic technique throughout the procedure and carefully monitored core temperature using a feedback-regulated heating pad set to maintain 37 ± 0.5 °C. Control animals received equivalent volumes of non-targeting AAV constructs delivered under identical conditions. Following surgery, animals were monitored closely for 72 h during recovery before proceeding with subsequent experimental procedures.

### EA intervention

EA intervention was administered to lightly anesthetized mice following our previous protocols [32]. Treatment targeted the bilateral "Fengchi" (GB20) and "Yanglingquan" (GB34) acupoints, which are well-established therapeutic points along the Foot Shaoyang Gallbladder Meridian with demonstrated efficacy in migraine management based on previous clinical and preclinical studies [19–21, 23]. GB20 was precisely localized 3 mm lateral to the midpoint between the ears, corresponding anatomically to the human depression between sternocleidomastoid and trapezius muscles, while GB34 was positioned near the knee joint at the anteroinferior aspect of the fibular head within the peroneal muscle group. Control animals received sham EA at non-acupoint locations approximately 10–15 mm above the iliac crest using identical electrical parameters to account for nonspecific stimulation effects (see Fig. S1B). Stainless steel acupuncture needles (0.16 × 7 mm) were inserted to a depth of 3 mm and connected to a Han’s acupoint nerve stimulator electroacupuncture device delivering stimulation at 1 mA intensity using 2/15 Hz dense-sparse wave frequencies for 15 min per session. Treatments were performed 30 min prior to inflammatory soup or saline injections and repeated every 48 h throughout the intervention period.

### Experimental Design

#### Experiment 1

To investigate the analgesic effects of EA, we employed an IS-induced migraine model, with sample sizes (n = 10/group for behavioral tests) determined by power analysis (G*Power v3.1, α = 0.05, power = 0.8). Forty-eight C57BL/6J male mice in Experiment 1 were randomly assigned to four experimental groups (n = 12/group): (1) control (saline injection + no treatment), (2) IS model (dural IS injection), (3) IS + EA (IS injection + EA treatment at GB20/GB34), and (4) IS + sham EA (IS injection + non-acupoint treatment). EA stimulates at GB20 and GB34 acupoints, while sham controls received identical stimulation at non-meridian locations (Fig. S1C). Given the repeated-measures behavioral design, a post hoc power analysis for the within–between interaction (repeated-measures ANOVA; effect size f = 0.25, α = 0.05, five repeated measurements) confirmed an achieved power of 0.97, indicating sufficient statistical power to detect group differences despite behavioral variability.

#### Experiment 2

Based on power analysis (G*Power v3.1, α = 0.05, power = 0.8), a sample size of n = 7 per group was determined for behavioral testing. Forty-two male C57BL/6J mice were randomly assigned to six experimental groups (n = 7/group) using a computer-generated randomization scheme: (1) IS (inflammatory soup only), (2) IS + AAV-*Cxcr5* (IS with *Cxcr5* overexpression), (3) IS + AAV-*Cxcr5*-NC + EA (IS with negative control virus + EA treatment), (4) IS + AAV-*Cxcr5* + EA (IS with *Cxcr5* overexpression + EA treatment), (5) IS + shRNA (IS with *Cxcr5* knockdown), and (6) IS + shRNA-NC (IS with scrambled shRNA control). All viral vectors were stereotaxically microinjected into the Sp5C region 3 weeks prior to dural cannulation surgery to allow for sufficient transgene expression (see Fig. S2C). Post hoc power analysis based on the repeated-measures ANOVA design (effect size f = 0.25, α = 0.05, five repeated measurements) yielded an achieved power of 0.86 for the within–between interaction, exceeding the conventional threshold of 0.80 and supporting the adequacy of the sample size.

### Behavioral testing

Von Frey filaments (mechanical) and hot plate/tail flick tests (thermal) are the most widely used methods in preclinical migraine research to assess tactile allodynia and thermal hyperalgesia, respectively.

### Mechanical withdrawal threshold

Mechanical allodynia was assessed using the up-down method with calibrated von Frey filaments (Stoelting, Wood Dale, IL) to measure the 50% face mechanical withdrawal threshold (FMWT) and hindpaw mechanical withdrawal threshold (PMWT) [32]. Prior to testing, mice were acclimated for 30 min in ventilated plexiglass chambers to minimize stress-induced behavioral variability. Baseline measurements were obtained before any dural interventions to establish normative values. Mice were acclimated restricted in a special experimental cage for another 30 min before the behavioral tests. Periorbital and hindpaw hyperalgesia were both measured to assess hypersensitivity in the migraine mouse model.

For periorbital testing, filaments ranging from 0.16 g were applied perpendicularly to the periorbital region until buckling occurred, maintaining consistent 2 s stimulation intervals. A positive response was recorded when mice exhibited clear avoidance behaviors (rapid head withdrawal, face wiping, or vigorous grooming). Hindpaw sensitivity was evaluated by applying filaments (starting with 1.0g) to the plantar surface for 5 s durations, with paw lifting, licking, or sharp retraction constituting valid responses. Each testing session included three trials per region with 5-min inter-trial intervals to prevent sensitization, and thresholds were calculated using the Dixon nonparametric method.

### Thermal withdrawal threshold

Thermal hyperalgesia represents one of the most clinically relevant manifestations of migraine-associated allodynia, frequently reported by patients during and between attacks. In preclinical studies, thermal nociception assays have become established metrics for evaluating migraine-related sensitization in rodent models.

### Hot plate latency (HPL)

Thermal nociceptive thresholds were evaluated using a standardized hot plate apparatus maintained at 52.5 ± 0.2 °C. Following 30-min acclimation in transparent plexiglass chambers, mice were individually placed on the heated surface, and latency to exhibit nocifensive behaviors (hindpaw lifting, licking, shaking, or jumping) was recorded with a 30-s cutoff to prevent tissue injury. Each animal underwent three trials at 15-min intervals to minimize stress-induced hyperalgesia, with the mean latency across trials calculated as the HPL. Testing was conducted in a quiet environment under consistent lighting conditions to reduce external variability.

### Tail-flick latency (TFL)

Thermal pain sensitivity was assessed using an automated tail-flick analgesia meter (IITC Inc., Woodland Hills, CA) set at 50 °C. Mice were gently restrained while radiant heat was applied to the ventral tail surface 1.5 cm from the tip. TFL was automatically recorded when the tail withdrew, with three trials conducted at 15-min intervals (10-s cutoff). The mean latency was calculated as TFL.

### Sample collection and processing

After CO_2_-induced euthanasia, brain tissues were promptly collected. The Sp5C regions were dissected on ice and immediately snap-frozen in liquid nitrogen for subsequent Western blot (n = 3/group) and ELISA (n = 6/group) analyses. For immunofluorescence studies, a separate cohort (n = 3/group) was deeply anesthetized and subjected to transcardial perfusion with 40 mL ice-cold 0.9% saline followed by 30 mL chilled 4% paraformaldehyde (PFA). Perfused brains were post-fixed overnight in 4% PFA.

### Immunofluorescence analysis

After fixation in 4% paraformaldehyde at 4 °C for 4 h, the tissues were transferred to 30% sucrose in PBS for dehydration at 4 °C overnight, until the brains sank to the bottom. Once dehydrated, the brain was embedded in OCT compound, oriented properly, and we used a cryostat to section the brain into 10-µm-thick slices. Then, we mounted the slices on slides coated with poly-L-lysine. The slices were blocked with 5% BSA for 60 min at room temperature and then incubated overnight at 4 °C with primary antibodies diluted in 1% BSA. On the next day, the slices/sections were then incubated for 2 h at room temperature in the dark with secondary antibody. Then the slices were washed and mounted on glass slides. For quantification, images from all slices/sections were taken under a 20 × or 40 × objective lens with a fluorescence microscope (Nikon Eclipse C1, Japan) and the fluorescence intensity of positive cells was calculated using ImageJ software. All the antibodies used are shown in Table S1.

### Western blotting

Sp5C tissues were homogenized in RIPA lysis buffer (Applygen) supplemented with protease and phosphatase inhibitors. Following centrifugation (13,000 × g, 10 min, 4 °C), protein concentrations in the supernatant were determined using a BCA assay. Protein samples (30 μg/lane) were denatured in loading buffer (Ding Guo) at 95 °C for 5 min, separated by 10% SDS-PAGE, and electrotransferred onto PVDF membranes. After blocking with 5% non-fat milk for 2 h at room temperature, membranes were incubated overnight at 4 °C with primary antibodies (Table S1), followed by species-matched HRP-conjugated secondary antibodies (1–2 h, RT). Protein bands were visualized using enhanced chemiluminescence (ChemiDoc™ XRS +, Bio-Rad) and quantified using ImageJ software (NIH).

### Enzyme-Linked Immunosorbent Assay (ELISA)

The protein levels of NR2B, substance P (SP), and pituitary adenylate cyclase-activating polypeptide (PACAP) in Sp5C tissue lysates were quantified using commercial ELISA kits (Cloud-Clone Corp, Wuhan, China), strictly following the manufacturer's protocols. Briefly, tissue homogenates were centrifuged (12,000 × g, 15 min, 4 °C) and supernatants were loaded in duplicate onto antibody-precoated 96-well plates. After 1 h incubation at 37 °C, wells were washed (3 × with PBS-0.05% Tween 20) and incubated with HRP-conjugated detection antibody (30 min, 37 °C). Following additional washes, enzymatic reactions were developed using TMB substrate (15 min, 37 °C in dark) and terminated with stop solution. Absorbance at 450 nm was immediately measured using a microplate reader (BioTek Synergy H1), with reference wavelength set at 630 nm.

### Real-Time Polymerase Chain Reaction (Real- time PCR)

Total RNA was extracted from 5–20 mg Sp5C tissue samples using RNA lysis buffer with mechanical homogenization (3 mm grinding beads), followed by chloroform/isopropanol precipitation. RNA concentration and purity were determined spectrophotometrically (NanoDrop 2000) and adjusted to 200 ng/μL. cDNA synthesis was performed using SweScript All-in-One RT SuperMix with integrated gDNA removal (Servicebio) according to manufacturer's protocol. qPCR reactions were carried out in 20 μL volumes containing 1 μL cDNA template, 0.2 μM gene-specific primers (Table S2), and SYBR Green master mix (Takara) on a Real-Time PCR Detection System. The thermal cycling protocol consisted of: (1) initial denaturation at 95 °C for 2 min; (2) 40 cycles of 95 °C for 15 s, 60 °C for 30 s, and 72 °C for 1 min; followed by (3) melt curve analysis (60–95 °C) to verify amplification specificity. All reactions were performed in triplicate with appropriate negative controls. Relative gene expression was calculated using the 2^−ΔΔCt^ method with β-actin as the endogenous reference.

### MicroRNA array analysis

#### Total RNA extraction

Small-RNA sequencing was conducted by a commercial service (CNKINGBIO, China). Total RNA was isolated from Sp5C brain tissue of migraine model and control mice (n = 3/group) using TRIzol reagent. RNA integrity was rigorously assessed through: (1) 1% agarose gel electrophoresis for degradation analysis, (2) NanoPhotometer spectrophotometry (A260/280 > 1.8; A260/230 > 2.0), (3) Qubit 2.0 Fluorometric quantification, and (4) Agilent Bioanalyzer 2100 evaluation (RIN > 8.0 required). Only high-quality samples proceeding to library preparation.

### Library preparation and sequencing

A total of 3 μg RNA per sample was used to construct small RNA libraries with the Small RNA Library Prep Set (NEB, USA). Size-selected RNAs (18–30 nt) were sequentially ligated to 3' and 5' adaptors, reverse transcribed with M-MuLV Reverse Transcriptase, and PCR-amplified with LongAmp Taq. Library fragments (140–160 bp) were gel-purified (8% PAGE) and validated using Agilent High Sensitivity DNA chips. Pooled libraries were sequenced on Illumina HiSeq 2500 (50 bp single-end reads; ~ 20 million reads/sample). Raw sequencing data are available at GEO (GSE281596).

### Prediction and enrichment analysis of microRNA target genes

Differentially expressed miRNAs were identified using DESeq2 (|log2FC|> 1, FDR < 0.05), followed by target gene prediction through an integrated analysis of two databases: TargetScan (https://www.targetscan.org/vert_80/) and miRanda (http://www.microrna.org/). The combined genes were subjected to functional enrichment analysis using the R package 'clusterProfiler' (version 4.10.1) [34], including Gene Ontology (GO) annotation (biological process, molecular function, and cellular component), KEGG pathway mapping, and disease ontology assessment, with statistical significance thresholds set at adjusted p-value < 0.05.

### Cell culture

Neuro-2a (N2a) mouse neuroblastoma cells were purchased from Procell Life Science & Technology Co., Ltd. (Wuhan, China) and cultured according to the supplier’s recommendations. Cells were maintained in Neuro-2a Cell Complete Medium (Procell, Cat. No. CM-0168), containing the proprietary supplements provided by the manufacturer. Cultures were maintained in a humidified incubator at 37 °C with 5% CO_2_.

### Cell transfection

Neuro-2a cells were transfected using Lipofectamine 3000 reagent (Invitrogen, USA) according to the manufacturer’s instructions. Briefly, cells were seeded into 6-well plates or 24-well plates and grown to approximately 60–70% confluence at the time of transfection. MicroRNA mimics, negative control oligonucleotides, plasmid constructs, or luciferase reporter vectors were diluted in Opti-MEM reduced-serum medium and mixed with Lipofectamine 3000 reagent to form transfection complexes. The complexes were then added dropwise to the cells and incubated under standard culture conditions. After 6–8 h of incubation, the transfection medium was replaced with fresh complete DMEM. Cells were harvested 24–48 h after transfection for subsequent analyses, including quantitative PCR, western blotting, or luciferase assays. Transfection efficiency was routinely monitored using control plasmids or fluorescence-labeled oligonucleotides, and only experiments with satisfactory efficiency were included in the analysis.

### Luciferase reporter assay

Neuro-2a cells were co-transfected with firefly luciferase reporter plasmids containing either the CXCR5 promoter region or the 3′ untranslated region (3′UTR) of target genes, together with a Renilla luciferase plasmid as an internal control. MicroRNA mimics or corresponding negative controls were co-transfected as indicated. At 24–48 h post-transfection, luciferase activity was measured using the Dual-Luciferase Reporter Assay System (Promega, USA) following the manufacturer’s protocol.

### Statistical analysis

Data are presented as mean ± SEM for behavioral experiments and as mean ± SD for all other quantitative data. Behavioral withdrawal thresholds were analyzed using repeated-measures analysis of variance (ANOVA), with time defined as the within-subject factor and treatment group as the between-subject factor. Group × time interactions were assessed to determine differential treatment effects over time. Sphericity was evaluated using Mauchly’s test, and Greenhouse–Geisser corrections were applied when appropriate. Post hoc comparisons following repeated-measures ANOVA were performed using Bonferroni-adjusted tests. All other data were analyzed using one-way ANOVA in SPSS v12.0 software (SPSS, Chicago, IL, USA). Post hoc comparisons were conducted using the Bonferroni test for homogeneous variances or the Tamhane test for heterogeneous variances. A p value < 0.05 was considered statistically significant.

## Results

### *EA attenuates migraine-like behaviors *via* CXCL13/CXCR5 signaling*

**Fig. 1:**
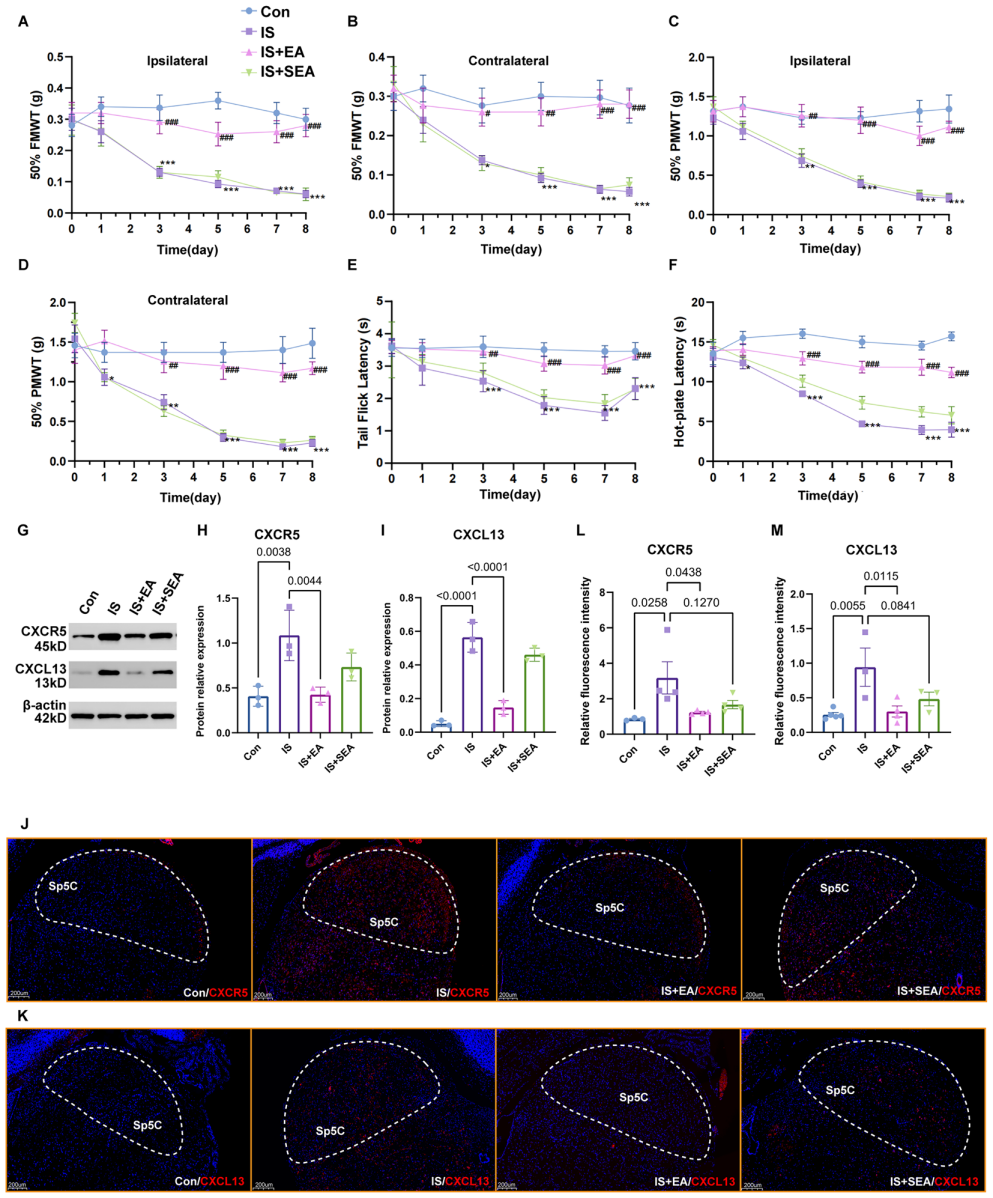
EA exerted analgesic effect and modulated CXCL13/CXCR5 expression after IS injection. **A**–**F** EA attenuated IS-induced mechanical allodynia and heat hyperalgesia and mediated expression of CXCL13/CXCR5. Behavioral changes were examined both ipsilateral and contralateral to the inflammatory soup injection site. Mean ± SEM. Two-way ANOVA, *p < 0.05, **p < 0.01, ***p < 0.001. vs. Con group, and #p < 0.05, ##* p* < 0.01, ###*p* < 0.001 vs. IS group. **G** Western blotting images and analysis showing the expression of CXCR5 **H** and CXCL13 **I** increased in Sp5C after IS-injection, and EA reduced their increased levels. Mean ± SD. One-way ANOVA, *p < 0.05, **p < 0.01, ****p < 0.0001. (H)R^2^ = 0.7888, (I)R^2^ = 0.9602. Immunofluorescence staining showed EA reduced up-regulated the number of CXCR5-positive **J**, **L** and CXCL13-positive **K**, **M** cells induced by IS, but not by SEA. Scale bar: 200 μm. Mean ± SD. One-way ANOVA, *p < 0.05, **p < 0.01. (L)R^2^ = 0.5238, (M)R^2^ = 0.6214. EA: electroacupuncture; IS: inflammatory soup

Behavioral assessments demonstrated that IS injection induced sustained mechanical allodynia (reduced 50% facial and paw withdrawal thresholds) and thermal hyperalgesia (decreased tail-flick and hot-plate latencies) at the ipsilateral and contralateral side of IS injection site from day 3 to day 8 post-induction (Fig. 1A–F, Table S3), confirming successful migraine model establishment as observed in previous studies [30]. EA at GB20/GB34 significantly reversed these nociceptive behaviors, while sham EA (SEA) showed no therapeutic effects (P > 0.05), indicating acupoint specificity (Fig. 1A–F).

At the molecular level, western blotting and immunofluorescence analysis revealed that IS challenge markedly upregulated both CXCL13 and its receptor CXCR5 in the Sp5C region (P < 0.05 vs control). Notably, EA treatment effectively normalized these elevations, while Sham EA showed no significant alteration compared with IS group (Fig. 1G–M), suggesting that the analgesic effect of EA is mediated through modulation of the CXCL13/CXCR5 chemokine pathway. These findings provide compelling evidence that the CXCL13/CXCR5 axis plays a crucial role in migraine-related nociception and represents a key target for EA intervention.

### *EA exerts anti-inflammatory and analgesic effects *via* CXCR5 modulation*

**Fig. 2:**
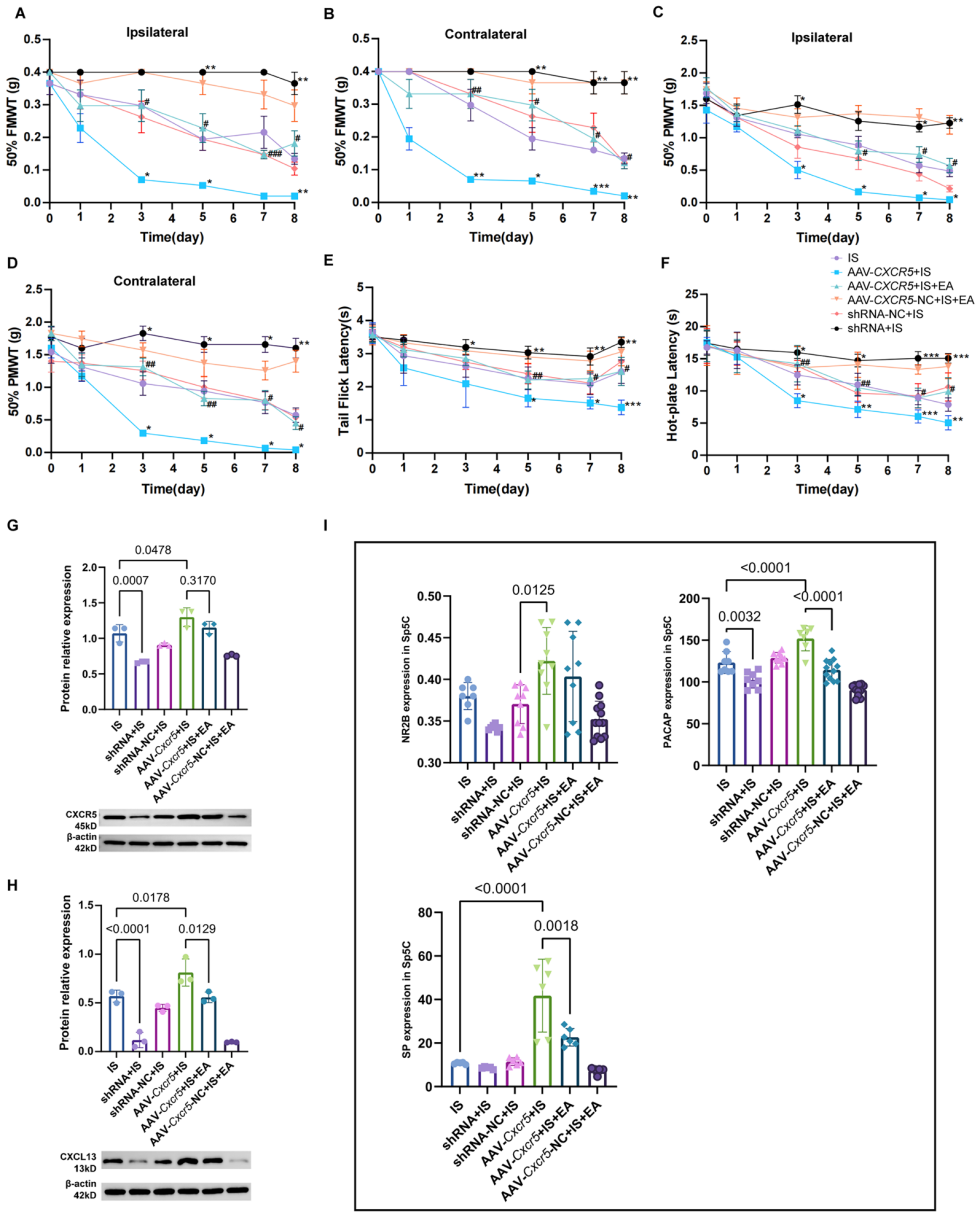
CXCR5 mediated EA analgesic effects by modulating neuron activity-related receptors, and neuropeptides. **A**–**F** EA alleviated the exacerbation of pain sensitivity induced by overexpression of CXCR5, and shRNA treatment increased the mechanical and thermal pain thresholds. Mean ± SEM. Two-way ANOVA, *p < 0.05, **  < 0.01, ***p < 0.001 vs. IS group, and #p < 0.05, ##p < 0.01, ###p < 0.001 vs. IS + AAV-*Cxcr5* group. N = 12. Western blotting images and analysis showing the expression of CXCR5 **G** and CXCL13 **H** increased in Sp5C after overexpressing CXCR5, and silencing CXCR5 gene reduced their increased levels. Mean ± SEM. One-way ANOVA, (H)R^2^ = 0.9148, (H)R^2^ = 0.9457. **I** ELISA analysis showed that AAV-Cxcr5 increased expression of NR2B, SP and PACAP, and CXCR5-shRNA decreased the expression of them, like the effect of EA. Mean ± SEM. One-way ANOVA. EA: electroacupuncture; IS: inflammatory soup

To elucidate the specific involvement of CXCR5 in EA-mediated therapeutic effects, we performed functional manipulation of CXCR5 expression in the Sp5C region. Viral vector-mediated overexpression of CXCR5 (AAV-*Cxcr5*) significantly exacerbated IS-induced thermal hyperalgesia and mechanical allodynia, whereas CXCR5 knockdown (AAV-*Cxcr5* shRNA) attenuated these nociceptive behaviors (Fig. 2A–F, Table S4). Consistent with these behavioral findings, molecular analysis revealed that CXCR5 knockdown normalized IS-induced upregulation of both CXCL13 and CXCR5 protein expression, while CXCR5 overexpression further enhanced their levels (Fig. 2G, H). Notably, EA treatment produced effects comparable to CXCR5 knockdown, effectively reversing nociceptive hypersensitivity and neurochemical alterations. Conversely, the therapeutic efficacy of EA was diminished in CXCR5-overexpressing mice, demonstrating functional dependence on this receptor. Complementary ELISA analysis of migraine-relevant neuropeptides showed that CXCR5 manipulation bidirectionally regulated NR2B, PACAP, and SP expression, while CXCR5 overexpression potentiated IS-induced elevations, while knockdown produced normalization similar to EA treatment.(Fig. 2I). The anti-nociceptive and anti-inflammatory effects of EA in migraine are mediated through targeted regulation of CXCL13/CXCR5 signaling in the Sp5C region.

### EA modulates neuroinflammation through CXCL13/CXCR5/ERK signaling

**Fig. 3:**
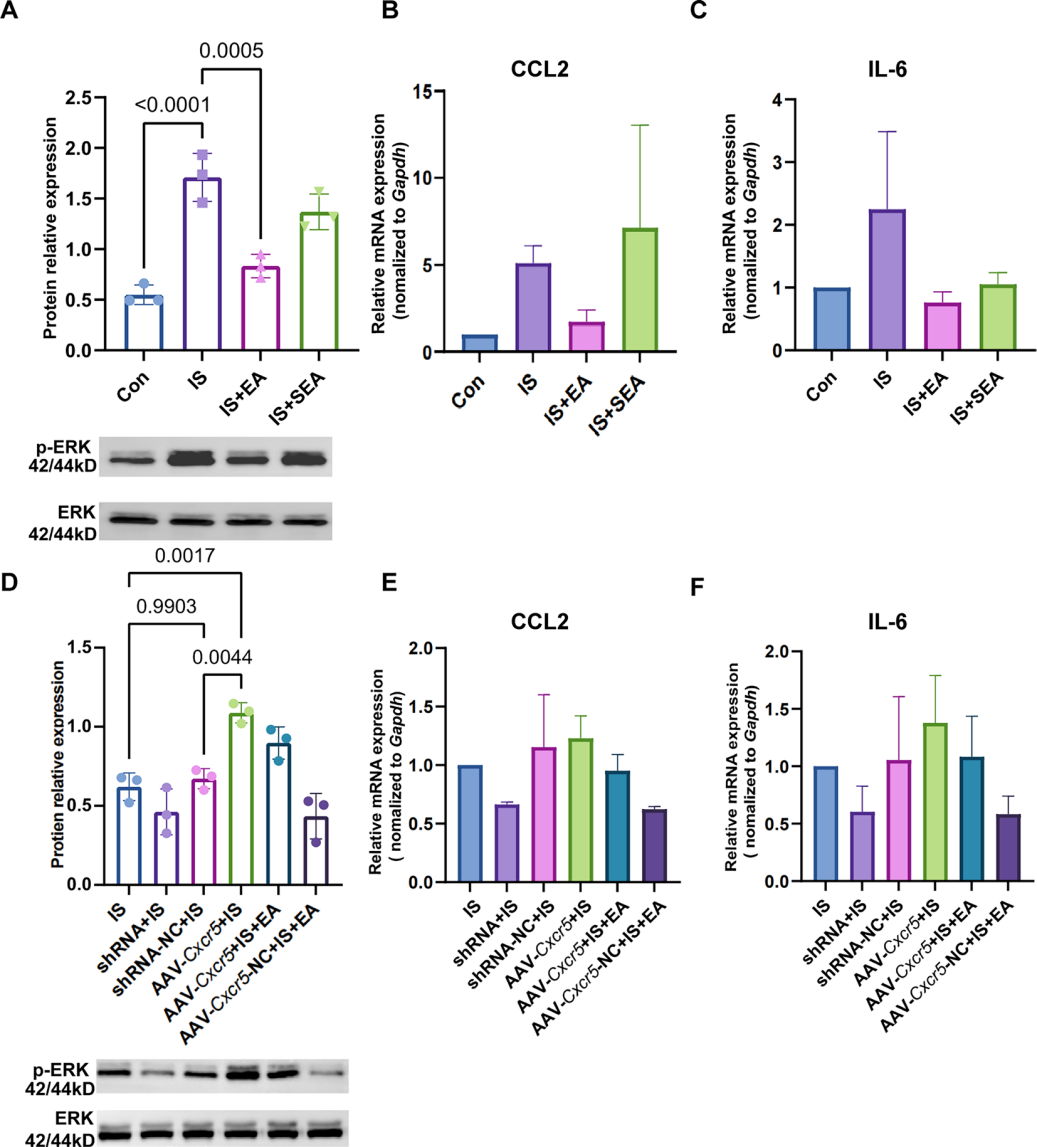
EA mediated neuroinflammation by CXCR5. **A** EA suppressed the increase in ERK phosphorylation caused by IS. Mean ± SD. One-way ANOVA, R^2^ = 0.8540. **B**, **C** IS significantly increased IL-6 and CCL2 mRNA expression, and EA treatment significantly decreased their up-regulation, but not by SEA. **D** EA suppressed the increase in ERK phosphorylation caused by AAV-CXCR5. Mean ± SD. One-way ANOVA, R^2^ = 0.8775. **E**, **F** Overexpression of CXCR5 led to an increased elevation of IL-6 and CCL2 mRNA levels induced by IS, while silencing the CXCR5 gene can achieve the same inhibitory effect as EA. Mean ± SEM. One-way ANOVA. EA: electroacupuncture

Further investigation into the molecular mechanisms revealed that EA exerts its anti-inflammatory effects by regulating the CXCL13/CXCR5/ERK signaling axis in migraine model mice, which is known to be an essential role in the pro-inflammatory pathway [35]. Western blotting analysis demonstrated that IS injection significantly increased phosphorylated ERK1/2 levels in the Sp5C region, while CXCR5 overexpression via AAV-Cxcr5 further amplified this ERK activation (Fig. 3A, D). The qPCR analysis revealed upward trends in *Il6* and *Ccl2* mRNA expression following IS induction consistent with their expected pro-inflammatory roles, yet these increases did not reach statistical significance (Fig. 3B, C, E, F). Importantly, EA treatment effectively counteracted these pathological changes, significantly reducing IS-induced phosphorylation of ERK1/2 and partly downregulating IL-6 and CCL2 expression. Notably, EA also attenuated the enhanced ERK activation and cytokine production caused by CXCR5 overexpression, demonstrating its capacity to modulate neuroinflammation through the CXCL13/CXCR5-ERK signaling pathway.

### Neuronal communication of EA's anti-inflammatory and analgesic effects

**Fig. 4:**
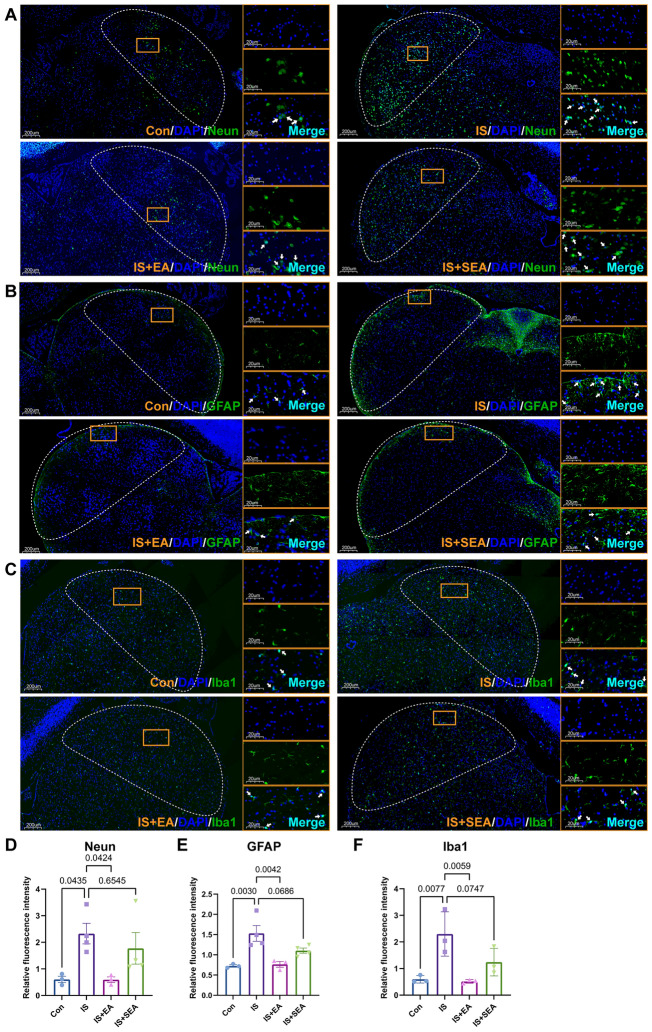
EA regulated neuron, astrocyte, and microglia population. **A**–**D** EA reduced distribution of neuron in IS group. Mean ± SEM. One-way ANOVA, R^2^ = 0.5566. **B**, **E** EA reduced distribution of astrocyte in IS group Mean ± SEM. One-way ANOVA, R^2^ = 0.7321. **C**, **F** EA reduced distribution of microglia in IS group Mean ± SEM. One-way ANOVA, R^2^ = 0.7560

**Fig. 5:**
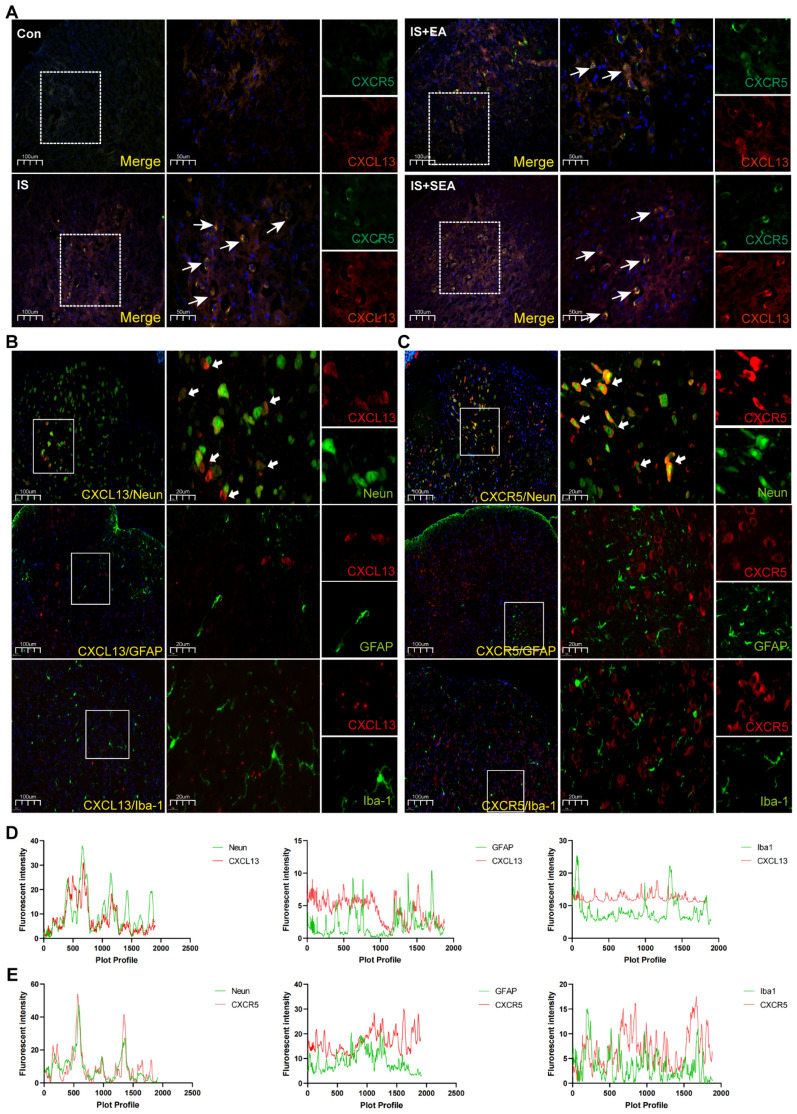
EA regulated co-expression of CXCL13/CXCR5. **A** Double immunostaining of CXCL13 and CXCR5 increased in IS group and decreased by EA. Scale bar: 100/50um. **B**, **D** Immunofluorescence staining showed the CXCL13 were primarily colocalized within neuron (NeuN positive), rarely within astrocytes (GFAP positive) and microglia (Iba1 positive) in the Sp5C site. **C**, **E** Immunofluorescence staining showed the CXCR5 were primarily colocalized within neuron (NeuN positive), rarely within astrocytes (GFAP positive) and microglia (Iba1 positive) in the Sp5C site. Scale bar: 100/20 µm. EA: electroacupuncture; IS: inflammatory soup

To investigate whether neuronal crosstalk mediates EA's effects through the CXCL13/CXCR5/ERK axis, we performed immunofluorescence analysis of cellular distribution patterns and CXCL13/CXCR5 co-localization in the Sp5C of migraine model mice. The immunofluorescence analysis revealed that IS induction significantly increased the distribution of neurons (Fig. 4A, D), astrocytes (Fig. 4B, E), and microglia (Fig. 4C, F) in the Sp5C, while EA intervention effectively suppressed this increase. SEA showed a potential effect but without statistical significance. In Fig. 5A, the chemokine CXCL13 and its receptor CXCR5 exhibited increased co-localization following IS infusion, predominantly in neuronal populations with minimal astrocytic involvement. In addition, CXCL13 mainly co-localized with neurons and had slight localization with astrocytes (Fig. 5B, D). CXCR5 also showed more co-localization with neurons (Fig. 5C, E). EA treatment effectively attenuated IS-induced cellular hyperreactivity and reduced CXCL13/CXCR5 co-expression, while SEA showed no significant effects. These findings demonstrate that EA's anti-inflammatory and analgesic actions in migraine are mediated through neuronal CXCL13/CXCR5/ERK signaling, with particular involvement of neuron-to-neuron communication pathways.

### Identification of microRNAs upstream of CXCR5 in EA-mediated regulation

**Fig. 6:**
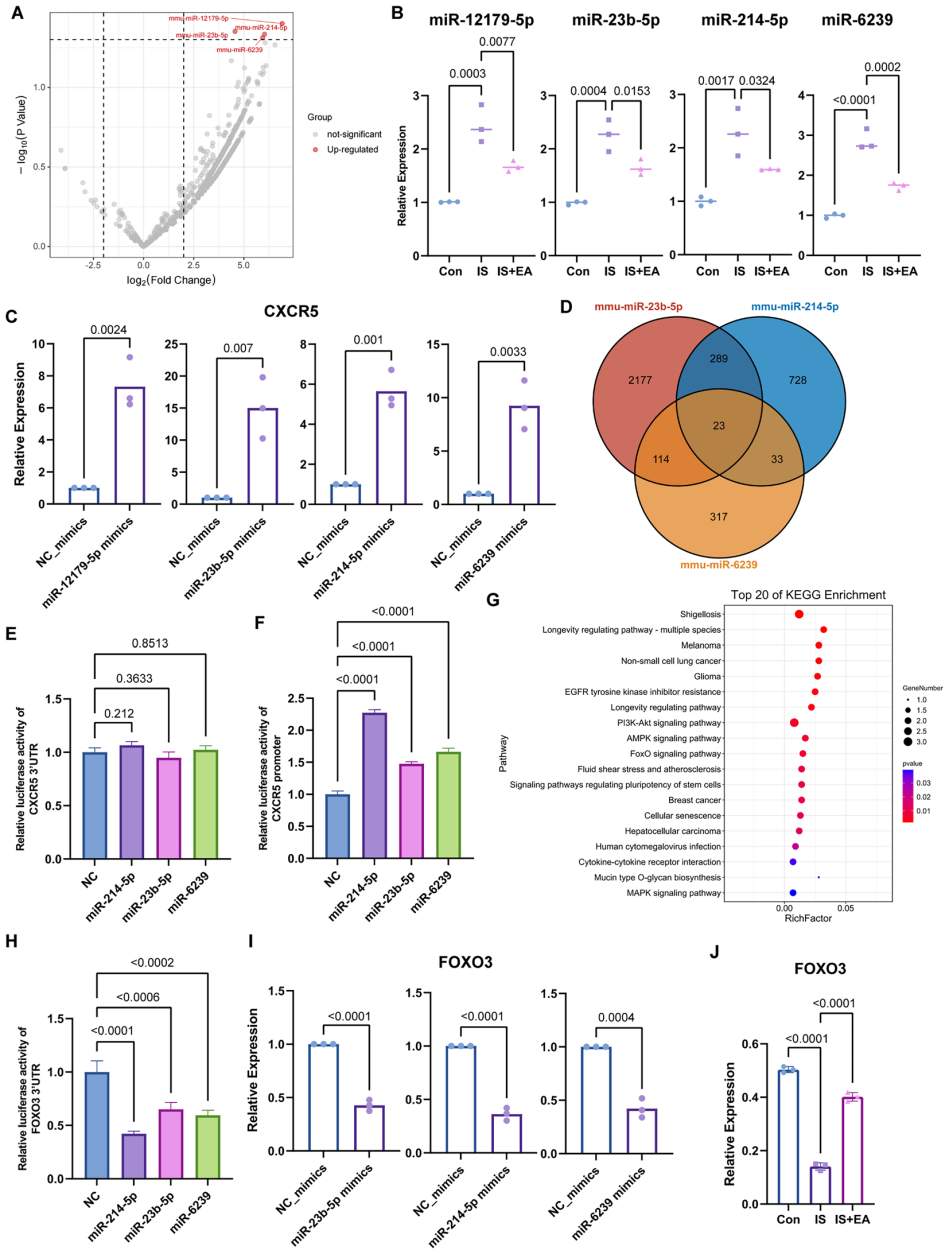
Identification of a miRNA–FOXO3–CXCR5 regulatory axis. **A** Volcano plot of microRNA expression profiles in the Sp5C region, showing differentially expressed microRNAs between experimental groups. **B** Quantitative PCR analysis of miR-12179-5p (R^2^ = 0.9155, Con vs. IS, p = 0.0003; IS vs. IS + EA, p = 0.0077), miR-23b-5p (R^2^ = 0.9204, Con vs. IS, p = 0.0004; IS vs. IS + EA, p = 0.0153), miR-214-5p (R^2^ = 0.8581, Con vs. IS, p = 0.0017; IS vs. IS + EA, p = 0.0324), and miR-6239 (R^2 ^= 0.9721, Con vs. IS, p < 0.0001; IS vs. IS + EA, p = 0.0002) expression in the Sp5C across the indicated groups. **C** Quantitative PCR analysis of CXCR5 mRNA expression in Neuro-2a cells following transfection with the indicated microRNA mimics or negative control (miR-12179-5p vs. NC, R^2^ = 0.9215, P = 0.0024; miR-23b-5p vs. NC, R^2^ = 0.8662, P = 0.007; miR-214-5p vs. NC, R^2^ = 0.9484, P = 0.001; miR-6239 vs. NC, R^2 ^= 0.9071, P = 0.0033). **D** Venn diagram showing the overlap of predicted target genes for miR-23b-5p, miR-214-5p, and miR-6239 based on TargetScan and miRanda analyses. **E** Dual-luciferase reporter assay assessing the effect of the indicated microRNA mimics on CXCR5 3′ untranslated region (3′UTR)–driven luciferase activity (R^2^ = 0.5997, miR-214-5p vs. NC, p = 0.212; miR-23b-5p vs. NC, p = 0.3633; miR-6239 vs. NC, p = 0.8513). **F** Dual-luciferase reporter assay evaluating the effect of the indicated microRNA mimics on CXCR5 promoter–driven luciferase activity (R^2^ = 0.9929, miR-23b-5p vs. NC, p < 0.0001; miR-214-5p vs. NC, p < 0.0001; miR-6239 vs. NC, p < 0.0001). **G** KEGG pathway enrichment analysis of the shared predicted target genes identified in **D**. **H** Dual-luciferase reporter assay examining the effect of the indicated microRNA mimics on FOXO3 3′UTR–driven luciferase activity (R^2^ = 0.9363, miR-23b-5p vs. NC, p < 0.0006; miR-214-5p vs. NC, p < 0.0001; miR-6239 vs. NC, p < 0.0002). **I** Quantitative PCR analysis of FOXO3 mRNA expression in Neuro-2a cells following transfection with the indicated microRNA mimics or negative control (miR-23b-5p vs. NC, R^2 ^= 0.9893, P < 0.0001; miR-214-5p vs. NC, R^2^ = 0.9885, P < 0.0001; miR-6239 vs. NC, R^2 ^= 0.9683, P = 0.0004. **J** Western blot analysis of FOXO3 protein expression in the Sp5C across the indicated experimental groups (R^2^ = 0.9945, Con vs. IS, P < 0.0001; IS vs. IS + EA, P < 0.0001). Data are presented as mean ± SD.

We performed microRNA sequencing of the Sp5C region in naive and IS-treated mice. Four microRNAs were identified as significantly differentially expressed (miR-12179-5p, miR-23b-5p, miR-214-5p, and miR-6239; P < 0.05, FDR < 0.05, fold change > 1.5. Fig. 6A, Table S5-9), all of which were upregulated in the model group. Quantitative PCR analysis of Sp5C tissue demonstrated that all four microRNAs were significantly upregulated in model mice, and that electroacupuncture treatment markedly reduced their expression levels (Fig. 6B). To explore the functional relationship between these microRNAs and CXCR5, Neuro-2A neuronal cells were used for in vitro validation. Overexpression of miR-12179-5p, miR-23b-5p, miR-214-5p, or miR-6239 led to a significant increase in CXCR5 expression (Fig. 6C). To identify potential intermediate targets, TargetScan and miRanda were employed for target gene prediction. As miR-12179-5p yielded no reliable predicted targets, intersection analysis was performed using predictions from miR-23b-5p, miR-214-5p, and miR-6239, resulting in 23 shared candidate target genes (Fig. 6D**, **Table 1). However, luciferase reporter assays demonstrated that transfection of these microRNAs caused no significant reduction in CXCR5 3′ untranslated region (3′UTR) luciferase activity (Fig. 6E), indicating that CXCR5 is not a direct post-transcriptional target of these microRNAs. We next assessed whether these microRNAs regulate CXCR5 at the transcriptional level. Using a CXCR5 promoter–driven luciferase reporter, we found that miR-214-5p robustly enhanced CXCR5 promoter activity, while miR-23b-5p and miR-6239 produced modest but consistent increases (Fig. 6F). These findings suggest that these microRNAs do not act through the CXCR5 3′UTR, but rather indirectly activate CXCR5 transcription, likely via repression of upstream transcriptional regulators.

**Table 1:** The venn results of target genes

mmu-miR-23b-5p	mmu-miR-214-5p	mmu-miR-6239	Set	Values	Count
TRUE	TRUE	TRUE	mmu-miR-23b-5p∩mmu-miR-214-5p∩mmu-miR-6239	Sit1, Cdk6, Igf1r, Ankh, Lhfpl4, Gbx2, Dst, Lpp, Oprm1, Cmtm4, Foxo3, Acvr2b, Fnbp1l, Tshz2, Fam179a, Slc25a29, Rad54l2, Il1r1, Prr14l, Hspa14, Galnt15, Epb41l5, Pou6f2	23
FALSE	TRUE	TRUE	(mmu-miR-214-5p∩mmu-miR-6239)∖(mmu-miR-23b-5p)	Aes, C1orf21, Sub1, Pik3c2a, Vgll4, N4bp3, Tub, Sostdc1, Calml3, Sstr2, St6galnac3, Fam43b, Prlr, Nyx, Stk40, Hbs1l, Pogk, Pbx1, Nfia, Dab1, Fxn, Cenpb, Arih1, Phc1, Mrps25, Osbpl5, Pde4dip, Rnf214, Ccdc73, Tmem179, Engase, Slc16a3, Timp2	33
TRUE	FALSE	TRUE	(mmu-miR-23b-5p∩mmu-miR-6239)∖(mmu-miR-214-5p)	Tro, Ca12, Pik3ip1, Gosr1, Hlf, Tbpl1, Tardbp, Tmod2, Galnt8, Sco1, Bach2, Tmem63b, Arhgap20, Arl1, Blmh, Letmd1, Itga6, Add2, Fstl4, Rhof, Srrm4, Zcchc14, Osr1, Kctd15, Cdc42ep3, Abi2, Prrc1, Arhgef40, Socs5, Pdcd6ip, C4orf32, Baalc, Tnks, Tbc1d8b, Ahrr, Mkl2, Pvrl3, Gls, Muc20, Minos1, Smad3, Znf704, Kpna4, Celf4, Lhx9, Dlk1, Pld1, Mbtps1, Nptn, Nkx2-1, Arel1, Man1a2, Fam212b, Scn3a, Pfkfb3, Smurf1, Shisa4, Mixl1, Prox1, Ate1, Impg1, Enox2, Lrrc7, Sgip1, Lcor, Pafah2, Otud3, Wnk3, Irs2, Kazn, Pcdh17, Fam219a, Ptchd1, Ndnf, Itsn1, Rfx3, Zic4, Gpr55, Mapk9, Cnp, Abcd3, Lynx1, Znf169, Scaf11, Pde1c, Col25a1, Tmem26, Dlgap1, Phf14, Prdm6, Samd12, E2f7, Pax7, Tanc2, Tspan14, Trove2, Zfp1, Ubn2, Phldb2, Klhl29, Scp2, Kiaa1211, Polr3g, Rp11-766f14.2, Nup160, Kcnj5, Cacnb4, Galnt2, Rgma, Xpnpep3, Frrs1l, Hoxb1, Grin2b, Kcnj6	114
FALSE	FALSE	TRUE	(mmu-miR-6239)∖(mmu-miR-23b-5p∪mmu-miR-214-5p)	Cyp51a1, Osbpl7, Fam13b, Cttnbp2, Adam7, Cbx5, Klhl20, Rnf125, Tax1bp3, Akap10, Ccdc86, Kitlg, Polr3b, Arpc3, Klhl18, Slc9a2, Nt5c1a, Slc19a2, Tcf21, Tbx2, Cat, Scrn1, Zc3h13, Acvr1c, Pla2g12a, Dstn, Hn1l, Tomm40, Gadd45g, Gse1, Nfatc1, Exoc4, Sdc1, Arfip2, Kras, Sort1, Usp37, Tank, Ier3, Rmdn3, Bcl2l10, Kif11, Nap1l1, Tmem206, Ankrd12, Slc4a1, Arhgap44, Nlrp1, Sc5d, Gabra4, Adam23, Sema3a, Apba1, Eif2b2, Erp27, Alg10, Sord, Fgf7, Npc1, Igfbp4, Trpc1, Fabp2, Cacna1b, Epha4, Zic1, Cfdp1, Hs3st3a1, Adamts5, Xpc, Znf286b, Bach1, Cacna1d, Olfml2a, Ppp1r9a, Znf276, S100b, Ube2q1, Znf281, En1, Cckar, Tmem169, Nek10, Cldn19, Mad2l1, Tnfrsf21, Rad54b, C12orf50, Hif1an, Ac007431.1, Scg5, Nudt21, Sdhaf2, Fut9, Mboat2, Prkce, Insm2, Pde7b, Alg10b, Lclat1, Tcap, Dsel, Lrch4, Arl10, Nefh, Zmynd8, Fbxo31, Znf507, C7, Znf436, Csrnp3, Pnma1, Pkm, Clip4, Ppie, C7orf41, Ece2, Diaph2, Ndufaf3, Plcxd3, Atp6v0a2, Rp11-480i12.4, Tacc1, Opcml, Fam83f, Rgs9bp, Btbd7, Dnajb2, Zcchc7, Sdc3, Cep63, Mettl24, Naaladl1, Mios, Gpt2, C5orf47, Slc37a3, Kcnk10, Adipor1, Ankrd55, Dcaf5, Nckipsd, Cdh5, Pbx3, Rap2c, Pcsk6, Adrb3, Nol10, Thsd4, Vat1, Slc25a46, Tat, Myo16, Dcx, Fn1, Bcl11b, Zbtb18, Ceacam19, Hyal3, Emp2, Pim3, Znf711, Mylk, Gramd4, Tsen15, Naa35, Ilf2, Lrp2bp, Slc39a13, Mlk4, Syt14, Dyrk3, Xcl1, Ddr2, Stx7, Kcnj9, Kcnj10, Gba, Zbtb7b, C6orf165, Wnt2b, Ube3d, Cox7a2, Ythdf1, Golga7b, Lgsn, Gpc4, Frat2, Gpr115, Podn, Exoc6, Cmpk1, Minpp1, Comtd1, Foxj3, Dbndd2, Fam129b, Vstm2l, Epb41, Ptafr, Slc44a1, Spin4, C20orf144, Ankrd26, Atp6v1g2, Zic2, Mthfr, Tnfrsf9, Hectd4, Rrbp1, Glipr2, Flrt3, Fam171a1, Aard, Itm2b, Id4, Sept8, Kiaa0226l, Prkd3, Tpd52, Nfib, Epha6, Rhbdf2, Pgbd5, Zfp82, Rbm33, Pou2af1, Maf, Mon2, Fbxl20, Ahsa2, Rtn1, Trim74, Mprip, Znf286a, Lrrc2, Ncald, Hoxa3, Fst, Mycl, Znf2, Add1, Mroh6, Ac003102.1, Rprd2, Phf21b, Triobp, Tbl1x, Wipf3, Rab1a, Mroh7, Ube2e3, Tmem182, Map2k4, Tp63, Magi2, Crtc3, Cask, Auh, Il1rap, Nkx2-5, Phactr2, Znhit6, Tfr2, Slc25a17, Exosc6, Esr1, Usp46, Ap1s3, Taf1d, Sema4d, Trim73, Gng4, Camk2b, Dapk1, C4orf36, Med12l, Rybp, Snx5, Fsbp, C9orf85, Lsamp, Rpl24, Phc3, Setd7, Inpp4b, Parm1, Mcidas, Ccdc160, Fam92a1, Lyn, Mcf2, Fli1, Sssca1, Ppfibp2, Ndufs3, Ube3b, Tbc1d30, Ankrd35, Mst1, Dnajb5, Akap3, Cpm, Lrp1, Papola, Fam227b, Tpm1, Chd9, Gan, Aipl1, Socs7, Dynll2, Kiaa1328, Tspan16, Ac138655.1, Oxnad1, Nudt3, Scoc	317
TRUE	TRUE	FALSE	(mmu-miR-23b-5p∩mmu-miR-214-5p)∖(mmu-miR-6239)	Nipal3, Akap11, Ctns, Lcp2, Tll1, C17orf85, Kiaa0141, Aqp2, Gtpbp1, Cbx7, Fermt1, Hoxa13, Wasl, Ccser2, Syngr2, Ufm1, Rab22a, Msto1, Lif, Hoxd9, Hoxd11, Bcl2l2, Gins2, Capn7, Zfyve20, Lin28a, Rcbtb1, Dab2ip, Gabbr2, Gnb5, Rbms2, N4bp1, Ppp1r12c, Itga9, Xrn1, Mgat4a, Map3k13, Tcf12, Rnf157, Rnf165, Kcnn3, Exoc6b, Fbxo25, Fam57b, Fer, Qtrtd1, Rab3c, Thy1, Marveld1, Unc5d, Wnt9b, Btg2, Cxcr5, Nacc1, Thap8, Tmem143, Nckap1l, Draxin, C1orf115, Gabrb1, Dnajc5g, Zdhhc3, Myo1a, Med1, Trpv3, Vstm2a, Mcc, Brd3, Prkcb, Agap1, Sik2, Mgmt, Atoh8, Ptpn9, Otud7a, Fnip1, Kcng4, Ppargc1b, Gga2, Cbx2, Heg1, Ppp1r8, Trib1, Rnf152, Krt80, Grina, Sertad2, Stx4, Samd4b, Edc3, Fbn1, Tollip, Amotl1, Znf318, Plxna4, Ppip5k2, Ahcyl2, Rnf185, Iqsec3, Rab11b, Alx4, Csf1, Flrt2, Colec10, Dlst, Col4a6, Bcl9l, Pcgf5, Larp1, Znf576, Prmt7, Rassf4, Xrra1, Rrp1b, Anapc1, Ldoc1l, Ankfy1, Dpp8, Ism2, Map2k3, Espnl, Ntn4, Mapt, C15orf62, Med22, Skap2, Tjp1, Camk2a, Gga3, Lmbr1, Pptc7, Git2, Lmx1b, Arsa, Sh3bp2, Rnf19b, Pax5, Nf1, Klhl14, Spn, Nrp2, Naa60, Dnajc5, Kiaa0753, Fam109a, E2f2, Tead1, Sh3bp5l, Cnst, Ryr2, Kiaa1614, Gpr161, Efna3, Chtop, Plekho1, Hspa12a, Gsto2, Ina, C10orf76, Ldoc1, Bag2, Opn5, Rab3b, Adamts13, Tmem53, Ncs1, Jph2, Mtf1, Fam102a, Spock2, Ago1, Ago4, Lhx6, Srpk1, Zdhhc18, C10orf126, Cenpp, Szrd1, Diras2, Shc3, Shisa7, Hs6st3, Ppp1r11, Gabbr1, Klf12, Lrrc10b, Rgp1, Cllu1, Camk1d, Fam169a, Ptcd2, Gsg1l, Tusc3, Lgi2, Olig2, Pou6f1, Akt2, Slc22a3, Dpy19l3, Mfsd6, Gtdc1, Grk5, Vti1a, Nme1, Mdm1, Ergic1, Hap1, Ccnjl, Adcy7, Spock1, Tcf7, Socs4, Vps13b, Polr3h, Gne, Slc38a9, Agpat6, Stk24, Arhgef12, Mbp, Tomm6, Ttc3, Garem, Cacna1c, Klhdc7a, Kiaa1671, Rbfox2, Slc8a1, Hic2, Aak1, Mgat5, Lrrtm4, Usp40, Wdfy4, Sp140l, Gosr2, Slc25a19, Synpo2, Tbl1xr1, Zbtb10, Rimkla, Tmcc1, Map6, Al354993.1, Serpina1, Psd4, Shisa6, Trim67, Ptpro, Mxra8, Dctn3, Cadm1, Tmem218, Stmn1, Pwwp2a, Sarm1, Clstn2, Ddi2, Kcna2, Ppp2r5d, Cep41, Arl15, Sncaip, Sept11, Slc35d1, Zfyve28, Smim3, Fxyd6, Ppp2r1b, Nxf1, Pianp, Fkbp5, Lyst, Tifab, Grm4, Daam2, Tshr, Rassf8, B2m, H2afj, Pias2, Cyb561a3, Lin7a, Xkr7, Cuedc1, Map2k6, Znf544, Trabd2b, Usp6nl	289
FALSE	TRUE	FALSE	(mmu-miR-214-5p)∖(mmu-miR-23b-5p∪mmu-miR-6239)	Ppp5c, Pigq, Rtn4r, Relt, Kcnq1, Otud5, Celsr3, St6gal1, Ntn1, Tram2, Tfap2c, Ppp1r13b, Siglec1, Mapk1, Cenpm, Dnal4, Cabp7, Tab1, Fam118a, Sall4, Txnl1, Kiaa1199, Tgm5, Bmf, Urgcp, Ykt6, Fbxl15, Obfc1, Rps6kb1, Hnf1b, Foxn1, Cyp27b1, Elk3, Crebl2, Gclc, B3gat2, Pcdh12, C5orf15, Pfkfb4, Tusc2, Rpl22, Wdr77, Fbxo30, Pkd2, Gsc, Mrc1, Serp1, Sorbs3, Twist1, Bhlhe41, Plcg1, Paip2b, Btn1a1, Cdkn1a, Pspn, Traf2, Strip2, Zc3h14, Ddx11, Rhobtb2, Dhx35, Pxdn, Fibcd1, Zc3h4, Tns4, Zswim4, Per2, Sbf2, Ctif, Edem1, Lpin1, Emp1, Itga7, Agap2, Rab11fip5, Epc2, Chst12, Tbrg4, Ralgps1, Gmpr, Ttbk1, Slco5a1, Nox5, Pak6, Kiaa1598, Dnajc13, Dirc2, C12orf49, Ac004381.6, Xylt1, Klf3, Traf5, Rimbp2, Ankrd13a, Prpf40b, Hcn4, Unc5a, Ltbp2, Slc11a2, Rpgrip1l, Cspp1, Alg1, Gnao1, Znf629, Chchd3, Rab36, Syp, Fam83e, Brd4, Taf1b, Spcs2, Selp, Lhx4, Il18rap, Srcin1, Sidt1, Cxcl9, Rasgrf2, Slc1a3, Ankrd32, Exosc7, Rps6ka2, Adap1, Hpx, Ccnj, Etnk1, Llph, Slc15a4, Zic5, Cbln3, B9d1, Prok1, Celsr2, Ildr2, Ensa, Golph3l, C1ql2, Slc20a1, Myh15, Cisd2, Phip, Bin3, Mal2, Fam173b, Pts, Slc7a11, Btbd11, Nrxn3, Qdpr, Farsb, Rnf219, Epg5, Tmem87b, Lpcat1, Cebpg, Fam105b, Fam167a, Gpr26, Vopp1, Ttll5, Cldn8, Pcdh1, Htr5a, Spata2, Irx6, Kiaa0895l, Zer1, Tmem199, Adar, Cyp11b1, Dcun1d1, Bcl6b, Megf6, Arl5a, Bmp10, Ccnyl1, Ccdc141, Stt3b, Il17rd, Bdh2, Wdr41, Kiaa0895, Sap30l, Slu7, Lmtk2, Pknox2, Bag5, Tmem132b, Hdgfrp3, Bean1, Sgk494, Pcsk9, Abr, C20orf196, Plp1, Il13, Nanp, Nmur1, Tacr1, Cd2bp2, Bcr, Stx18, Bmp1, Cep120, Has3, Nptx1, Axin2, Suclg2, Taok2, Fam192a, Klrc4, Klk10, Cped1, Cyb561d1, Mis18bp1, Dcaf7, Muc13, Rec8, Sh3pxd2b, Fbxo45, Speg, Vwc2l, Aggf1, Cyfip1, Dvl3, Chmp7, Dnajc25, Acad10, Csnk1d, Anp32e, Supt6h, Cd55, Fam71f1, Ctc1, Metap1d, Zfp91, Kiaa2018, Ttc9c, Snapc5, Zbed2, Prom2, Fbxo46, Slc5a10, Vps39, Cyfip2, Mrpl43, Etv4, Ush1g, Mrfap1l1, Kiaa1549l, Gpr137c, Ca13, Bptf, Wdfy3, Cyp11b2, Pipox, Hsf5, Plxnd1, Nupr1, Mllt6, Rab11fip4, Traf7, Ift80, Leng8, Noc2l, Timm22, Galk2, Ubald2, Ffar3, C16orf72, Hexdc, Caln1, Plb1, Gpr39, Gnb1l, Pex26, Mrto4, Tuba8, Cdca2, Hhipl1, Mrc1l1, Slc25a10, Tmem106a, Limk2, Setd3, Glul, Srek1, Nudt17, Krtap10-11, Fmnl3, Pitpnc1, Znrf1, Klhdc10, Hm13, Tnrc6b, Tnrc6c, Znf202, Zxdc, Ap3s2, Stard7, Ttc33, Akap1, Phf20l1, Eme1, Slc12a4, Palm, Vwa1, Tpst2, Eif4ebp1, Fam184a, Usp9y, Mltk, Oasl, Gjb4, Gab2, Tspan18, Scxb, Insig1, Lin54, Cpsf7, Znf746, Golgb1, Scxa, Cfl2, Nanos2, Rhbdd1, Napepld, Ifnar2, Lmx1a, Ctd-2228k2.5, Sox12, Sdhc, Srsf10, B3galtl, Ash2l, Nos1, Fam166a, Hdac4, Arid1b, Kcnip2, Grin1, Camk2g, Ccdc37, Acaca, Arpc5l, Cdrt1, Adra1a, Ak5, Far1, Gabpa, Rabl2b, Slc25a26, Lrrc20, Cab39l, Trappc3l, Fbrs, Tmprss11f, Ppp4r2, Rspo3, Nhej1, Med27, Dchs2, Flnb, Mier1, Asb13, C17orf102, Nat9, Aqpep, Elk4, Trdmt1, Arnt, Gulp1, Fgf1, Trpv6, Nup214, C1orf116, Itgb1bp1, Arpc5, Trrap, Kctd18, Ergic2, Uap1l1, Man2a2, Pea15, Tppp, Znf148, Tmem239, Lrrtm3, Mocs2, Pax9, Sepn1, Astn1, Fam19a3, Nos1ap, Ptgs1, Heatr1, B3galnt2, Irf2bp2, C1orf95, Dusp10, Gpatch2, G0s2, Plxna2, Serac1, Dstyk, Adora1, Gpr37l1, Mthfd1l, Mr1, Xpr1, Zbtb37, Adamts4, Slamf6, Sema4a, Dap3, Lce2c, Bub3, Znf687, Scnm1, Ca14, Cd160, Gstm5, Tlx1, Gabre, Cd99l2, Lrrc8c, Hpse2, Pi4k2a, Opalin, Entpd1, Ppap2b, Bsnd, Dhcr24, Dmrtb1, Pten, Pomgnt1, Nup188, Rims4, Dnajc9, Sytl4, Srpx2, Foxp4, Chst3, Fpgs, Ankmy1, Ak2, Yars, Phka1, Kpna6, Fabp3, C6orf106, C10orf53, Lrrc18, Susd1, Syt15, Man1c1, Cxcl12, Bmpr2, Ephb2, Tfdp1, Bud13, Padi1, Fbxo42, Col4a1, Prune2, Fbxo44, Kat5, Ubd, Cnnm4, Casc10, Tmem252, Fam196b, Cln5, Gpr153, Pcsk2, Tmem8b, Blace, Lig3, Ucma, Haus5, Lyrm7, Tmem170b, Trim36, Nrep, Tslp, Phka2, Ccdc85c, Usp3, Znf768, Hdac7, Bnc2, Kiaa1644, Igf2, Stk35, Vldlr, Smim21, Ccdc174, Tyrp1, Wiz, Tmem132d, Sptb, Klc1, Sqstm1, Glb1l2, Col23a1, Sprtn, Tp53bp2, Xrcc5, Fam117b, Grm1, Ccdc57, Nudt14, Ric8b, Rabl2a, Bcl2l11, Cnot6, Abtb1, Grk6, Slc29a1, Smarcc2, Brd7, Fam13a, Hyal1, Cldn3, Mapk10, Tp53i11, Purb, Vapb, Gli3, Ppara, Mob1a, Wdr72, Naga, Coch, Ube2g1, Ngdn, Repin1, Bloc1s5, Zbtb44, Ccdc41, C17orf67, Cdh7, C15orf38-ap3s2, Abcg1, Dpysl3, Kdm2a, Sptlc3, Aifm3, Ldlrad4, Cecr6, Kremen1, Mb, Elfn2, Ino80d, Rsph10b2, Nolc1, Rsph10b, Gpsm2, Ispd, Wdr43, C22orf29, Chrna2, Exd2, Rad21l1, Tet3, Cep19, Lysmd4, Nub1, Map4k4, Spag16, Prkca, Prdm2, Ss18, Znf609, Hla-dmb, Fam19a2, Rp11-195f19.5, Tmppe, Znf740, Atf7, Ncaph2, Clec2l, Rpl23a, C7orf49, Rpl7l1, Mepe, Wdr5, Eif4e3, Gatsl2, Parp11, Mro, Plekhm1, Creb1, Akirin1, Fbxo10, Arpc1a, Tmem51, Ccdc158, Cldn4, Gfap, Prdx2, Ac012215.1, Tars2, Gfra1, Slc46a1, Slc48a1, Cish, Myo5c, Mcfd2, Kcnk2, Fgf12, Flj00104, Lrrc34, Pacs2, Tecpr1, Ppfia4, Ppard, Arsg, Ascl5, Cxorf40a, Tmem151b, Sgca, Dse, Slc4a8, Dgki, Slc6a11, Cftr, Slc6a6, Pdxdc1, Catsper4, Fgf5, Scube2, Zbtb47, Dpcr1, Syne1, Rwdd1, Srprb, Errfi1, Hoxb3, Iah1, C1orf52, Rp11-73m18.2, St6galnac5, Efcab10, C6orf89, Rxra, Rcan1, Rgl2, Tmem167a, Pcp4l1, Znf667, C4orf21, Sepw1, Xrcc4, Larp1b, Gstcd, Fbxl8, Tenm2, Kcnk9, Dnm3, Ppil6, Atox1, Rnf130, Cacna1e, Znf585b, C1qtnf5, Dpf2, Fau, Ttc39a, H2afx, Fam118b, Calcb, Rp11-192h23.4, Pld5, Frmpd1, Pik3cd, Srrm1, Tmem258, Ac010336.1, Fetub, Cyld, Tlr5, Ccdc132, Podxl, Cdhr3, Asph, Dpf3, Gcfc2, C15orf37, Rassf3, Crb1, Ptpn14, Tomm5, Tasp1, Ac132186.1, Znrf3, Mybpc3, Cherp, Rp11-187e13.1, Wdr25, Mfrp, Lyrm5, Ppp2r5c, Wibg, Det1, Rp11-210m15.2, Secisbp2l, Eif2ak4, Tmem178b, Kiaa0513, Ctdnep1, Adcyap1, Cndp2, Rnf43, Sap30bp, Kank2, Polr2j2, Prcd, Fam20a, Al009178.1, Al031666.2, Lrrc4b, Dkfzp434h0512, Ac007375.1, Ppp1r26, Fxyd3, Hebp2, Zbtb8b	728
TRUE	FALSE	FALSE	(mmu-miR-23b-5p)∖(mmu-miR-214-5p∪mmu-miR-6239)	Fbxo38, Sox18, Foxn2, Slc4a4, C2orf88, Vwc2, Papln, Slc26a9, Ccng1, Nbr1, Mknk1, Mgat3, Armcx3, Sh2b3, Spg7, Bri3bp, Insr, Tmem248, Taf1c, Pmepa1, Taf9b, Cep97, Pdpk1, Pou2f2, Serinc3, Ascc1, Foxd4l3, Ppcdc, Tm7sf3, Ube2d3, Mafk, Atg5, Nt5c2, Dicer1, Hoxa9, Slc35e4, Cldn18, Wnt8b, Susd4, Psma8, Osgin1, Casz1, Dyrk2, Spry4, Rufy4, Npffr2, Iyd, Cdc42, Mrrf, Mcph1, Eng, Rsc1a1, Ets1, Thumpd3, Rad51d, Fermt3, Ppl, Ganab, Rab5c, Mtmr2, Camkk2, Dzip1, Kcnq4, Zfyve26, Mkks, Nup50, Kirrel2, Smarce1, Lepr, Smarca2, Cyth3, Ptpn11, Atp2b2, Ass1, Ceacam1, Zc3h7b, Mkln1, Abhd2, Prok2, Nsfl1c, Rfxank, Ywhab, Sept6, Crkl, Znf112, Mef2a, Nfat5, Tyw5, Ero1lb, Hkdc1, Lmo3, Acap3, Snd1, Pgap1, Glyctk, Fmod, Kif16b, Net1, Asgr2, Htt, Srgap1, Begain, Trpc7, Foxn4, Nhp2l1, Ap3d1, Ano1, Syt17, Erc1, Strn3, Mlh3, Mxra7, Ube2i, Atp5g1, Zgpat, Btg4, Rp11-366l20.2, Ccdc25, Fam180b, Pcyt1b, Pbrm1, Sptbn1, Bicd2, Adipor2, Gm2a, Sfpq, Col9a1, Galp, Acsl3, Homez, Dnajc14, Tmem170a, Pign, Brpf3, Asap1, Atp2b4, Grm7, Creb5, Mafg, Aig1, Sema4f, Tll2, Fam3b, C15orf57, Osbpl2, Gmfb, Wdr55, Armc8, Arhgap19, Entpd4, Ttc37, Ac079612.1, Gk2, March5, Pde3a, Pik3cg, Cmtm5, Slamf7, Fgfr2, Arap1, Plcg2, Rpe, Cplx2, Zranb1, Fbxl17, Slc6a12, Dda1, Sulf2, Atg2b, Dmbx1, Tpk1, Ssh1, Aph1a, Topors, Fbxl22, Nav2, Ppp3cb, Fut8, St7l, Gypa, Scyl2, Scube1, Sez6l, Fam107a, Pcdhb16, Lrig2, Iqgap3, Capza2, Atp1a2, C9orf114, Mt-nd4l, Ssbp3-as1, Nckap1, Tnni1, Tgm2, Ppef1, Muc2, Ipo9, Arhgap11a, St6gal2, Pcdh11x, Cntnap2, Cyb5r3, Grin3a, Spryd7, Msrb1, Pnma5, Mt-atp6, Ino80, Sfmbt2, Itsn2, Asb12, E2f6, Znf652, Fan1, Ahctf1, Kif26b, Efcab2, Akt3, Opn3, Slc35f3, C1orf198, Ccsap, Rhou, Gpr31, Enah, Fgfr1op, Pde10a, Taf1a, Hlx, Marc1, Slc30a10, Smyd2, Atf3, Sertad4, Dynlt1, C1orf147, Rab7l1, Nucks1, Slc45a3, Lemd1, Nuak2, Syt2, C6orf211, Phlda3, Cacna1s, Cdc73, Rnf2, Fam129a, Epm2a, Colgalt2, Utrn, Rgs16, Rnasel, Ier5, Heca, Serpinc1, Prrx1, Pou2f1, Uck2, Fcgr3b, Nr1i3, Arhgap30, F11r, Cd244, Cd84, Vangl2, Dcaf8, Cadm3, Spta1, Etv3, Insrr, Arhgef2, Hey2, Efna4, Atp8b2, Nus1, Ros1, Rab13, Nkx6-2, Fam26e, Tspyl1, Lama4, Clrn3, Adam12, Cdk19, Cep57l1, Ctss, Nr2e1, Pdss2, Qrsl1, Otud7b, Grik2, Sim1, Acp6, Klhl32, Adrb1, Rragd, Tectb, Gdap2, Fam46c, Rab39b, Igsf3, Fundc2, Slc22a15, Slc16a1, Kcnd3, Sorcs3, Prss35, Neurl, Neurl1b, Amigo1, As3mt, Sufu, Actr1a, Opn1mw2, Opn1mw, Kiaa1324, Vav3, Ntng1, Gmeb2, Slc30a7, Slc35a3, Dusp9, Poll, Kazald1, Gclm, Chrna4, Pnma3, Gfi1, Ogfrl1, Aff2, Mrgbp, Pkn2, Lmo4, Mcoln2, Ctbs, Fhl1, Lphn2, Gipc2, Mtg2, Mbnl3, Acadm, Frmd7, Fpgt, Elovl5, Frat1, Leprot, Itgb3bp, Alg6, Ocrl, Plce1, Enpp5, Cdc5l, Ffar4, Nfatc2, Npdc1, Bcas4, Il13ra1, Inpp5e, Dnlz, Znfx1, Nacc2, Prex1, Surf2, Pak3, Vegfa, Cdhr1, Dydc2, Mat1a, Ppapdc3, Aif1l, Ipo13, Artn, Al391421.1, Gpr107, Pltp, Hyi, Rbm41, Ermap, C1orf50, Hivep3, Rims3, Tp53tg5, Vcl, Cap1, Stk4, Urm1, Heyl, Ubr2, Taf7l, Tox2, Rragc, Pou3f1, Frs3, Inpp5b, Rpa4, Dpm2, Ptprt, Ago3, Sgpl1, Chd6, Sh2d3c, Tacr2, Pgk1, Traf3ip1, Zdhhc15, Glo1, S100pbp, Tbc1d22b, Gigyf2, Manbal, Txlna, Crb2, C2orf72, Acrc, Srsf3, Nkain1, Reep3, Ado, Ythdf2, Gmeb1, Rab14, Bicc1, Fam76a, Asah2b, Spdef, Tnfsf15, Nlgn3, Nfs1, Rgs3, Slc31a1, Snx30, Gprin2, Ldlrap1, Gdf5os, Clic4, Alox5, C1qc, Ikbkap, Rxrb, Abca1, Oxld1, Ppp3r2, Tp53inp2, Invs, Eif2s2, Notch4, Pla2g2d, Raly, Cbfa2t2, Apoa1, Kdm5c, Rcc2, Scn2a, Cobll1, Zbtb12, Dnmt3b, Rp11-114h20.1, Clcnkb, Kif3b, Ddah2, Shroom4, Clcn5, Foxp3, Ccdc93, Ppp1r10, Gnl1, Suv39h1, Gcnt1, Tbc1d25, Gpr180, Dffa, Tmem2, Cnnm3, Kif1b, Klf9, Ptar1, C20orf26, Crnkl1, Tgoln2, H6pd, Foxd4l4, Foxd4l6, Foxd4l2, Uchl3, Tbc1d4, Hif3a, Naa40, Slc17a4, C11orf83, Atg9b, Arhgap26, Cd177, Tbc1d31, Gba2, Ajap1, Ext1, C10orf111, Tp73, Tmx2, Ccl3l3, Fam213b, Dcdc2, Pank4, Fam214b, C2orf91, Hao1, Rad50, Crls1, Prr5l, Cxorf21, U2af1l4, Il1rapl1, Dnajc15, Gfod1, Vwa8, Nedd9, Kcnc1, Wbp4, Snx2, Eif1ax, Fosl2, Kifc3, Arrdc2, Slc35b3, Cyp1a1, Dsp, Nbea, Cdkl5, Nhs, Ift74, Txlng, Col4a3bp, Slc25a51, Zbtb4, B3galt5, Frmpd4, Pde4a, Slc7a1, Frem1, Nln, Sgtb, Kif2a, Itpr2, Serf2, Itpripl2, Tec, Lzts1, Cd274, Zzef1, C5orf51, Atp6v1c2, Slitrk4, Cys1, Ppip5k1, Srxn1, Dnajc21, Spata13, Onecut3, Zcchc3, Rnf215, Tas2r1, Sema5a, Foxd4, Dok6, Gjb2, Zfy, Stag1, Ralbp1, Eogt, Znf197, Trim71, Srgap3, Pip5kl1, Coro6, Dcp2, Dennd5b, Rnf20, Ptprn2, Gxylt2, Faxc, Ccdc88c, Nin, Pcnxl4, Ndufa3, Cacng7, Klk5, Znf180, Znf233, Hdlbp, Sh3bp4, Ttll4, Boll, Calcrl, Bdh1, Mb21d2, Tirap, Cdon, Veph1, Hmbs, Nt5dc3, Mybpc1, Ablim1, Ikbip, Steap3, Ptgfrn, Nrf1, Caps2, Kcnc2, Plxna1, Aldh1l1, Tsga10, Cog5, Rab24, Srpk2, Mfap3l, Wdr52, Mrpl19, Syt12, Ca7, Elmsan1, Cnpy3, Sv2b, Spry1, Akap13, Elovl6, Il16, Nrg2, Slc9b2, Dnaja4, Rcn2, Cdc23, Kif20a, Dak, Psap, C10orf54, Snca, Msantd3, Daam1, Znf365, Celf1, Psmb9, Dido1, Calml4, Atp2a2, Asah2, Specc1, Ulk2, Ext2, Il21r, Znf287, Bmp7, Mlc1, Ntrk2, Kcng1, St8sia1, Foxb1, Xirp1, Acbd5, Myd88, Snx10, Thrb, Ric3, Stxbp6, Gapt, Cldn6, Cpne6, Itga4, Hrh1, Lrrc57, Arpc4, Pla2g4f, Apol3, Galnt9, Jhdm1d, Ten1, Col6a2, Polr3d, Kiaa1211l, Tango2, Mblac1, Bcl2, Adam22, Pax1, Atl3, Col13a1, C17orf103, Ac007952.5, Cx3cr1, Jmjd1c, Smim11, Rbm12b, Hmgb1, Dlg4, Pla2g4e, Fam134b, Mapk8ip2, Runx3, Sirpa, L3mbtl4, Fam69c, Dleu7, Dscam, Greb1l, C9orf152, Hmx1, Prkcz, Nfasc, Pex13, Pde7a, Rapgef5, Mark2, Ano7, Rasgrp3, Akap12, Drp2, Fam150b, Gck, Dpp6, Hoxd12, Fgg, Apol4, Mmd2, Papolb, Hdac9, Ncor2, Cav1, Grhl1, Erbb2, Smcr8, Pard3b, Hipk2, Cbx6, Gucd1, Tfip11, Ica1, Xkr9, Hepn1, Tbx20, Foxp2, Lrrc29, Mogs, C17orf104, Ppp1r1c, Fam168b, Pcdh20, Ly6g6e, Snx13, Tmem237, Ipo11, Stk16, Lypd6b, Kcmf1, Tbc1d10b, Map3k2, Prpf40a, Plin2, Tmem25, Erbb3, Ptpn3, Rbm14-rbm4, Dazap2, Mov10, Exoc5, Ckmt1b, Hhat, Srgap2, Sprn, Scn5a, Sf3b1, Dnah3, Mum1, Abcc12, Knstrn, Smim13, Musk, Grpel2, Satb2, Rbm18, Rgs21, Frmd5, Grm5, Limk1, Ptprj, Luzp1, Lrtomt, Apoc4, Psma1, Bivm, Atxn7l1, Il6, Arrb1, Sbno1, Znf277, Gpr113, Perp, Ankrd34c, Atg2a, Ddn, Dio2, Nsl1, Rnaseh2b, Pomt1, Peg3, Fam120aos, Tmbim6, Chd7, Amer3, Cmtm3, Naa38, Rnf216, Hmga2, Ksr2, Igsf11, Myocd, C3orf18, Tmem185b, Tmem233, Arhgef28, Znf234, Sgsm2, Trpc4, Rnf5, Fgd4, Atxn1l, Dock4, Casp16, Ezh1, Nek11, Mical3, Abcf3, C1qtnf7, Gca, Calcoco1, Ip6k2, Cstf3, Kansl3, Tmprss11bnl, Ern1, Ctnna3, Slc13a5, Vipr1, Mgll, Rfc3, Sumf2, Dlx3, Kif5c, Sept7, Sgcd, Asxl2, Fchsd1, Mapre2, Sesn1, Glyr1, Gats, Ac106017.1, Nlrc5, Rarb, Parn, Adarb1, Nutm1, Cyhr1, Phf20, Bdnf, Disc1, Cisd3, Ccbe1, Pkmyt1, Kiaa1549, Fmo2, Ac006946.15, Al589765.1, Paqr8, Itfg3, Dcc, Znf516, Gdpd5, Dusp27, Ctdspl, Ampd3, Hnrnpu, Ints2, Smim1, Mlph, Slc25a36, Pik3r5, Plch1, Pla2g6, Zc4h2, Rp11-126k1.2, Rasal2, Dock9, Lmna, B3gnt9, Tmem120b, Ears2, Plch2, Nkx1-2, Ip6k3, Asb15, Ppp1r3e, Myh11, Unc5c, March8, Mtmr8, Arhgap19-slit1, Tjp2, Lilra1, Rell1, Etv3l, Ppih, Sypl1, Iffo2, Tbata, Sun1, Dthd1, Cd8a, Znf853, Mttp, Tyw3, Cnep1r1, Sulf1, Pxn, Cds2, Ptpn22, C3orf55, Xxbac-bpg32j3.20, Zbtb20, Pfdn6, Hltf, Wwtr1, Dnajc24, Cmc1, Ccdc74a, Clasp2, Rgs1, Rbm5, Golim4, Rp1-170o19.20, Zeb2, Fto, Selt, Wnt5a, Atpaf2, Nceh1, Fam120c, Yme1l1, Timm23b, Ezh2, Morn4, Gmppb, Ppp2ca, Iqcj, Vps52, Surf4, Calca, Alkbh6, Dnase1l3, Nr6a1, Rp5-850e9.3, Rhno1, Rwdd2b, Fam199x, Satb1, Dbnl, Ebp, Asap3, Fam163b, Lrrc55, Ppm1l, Pgam5, Tfdp2, Etf1, C5orf28, Usp51, Sh3tc2, Bend4, Pkd2l2, Ido2, C5orf20, Fryl, Megf10, Parp8, Ankrd50, Cnot6l, Timm8b, Ac005003.1, Ly75-cd302, Adamts12, Fam135a, Klhl3, Npy2r, Kctd16, C7orf73, Pacrgl, Rims2, Sorcs2, C5orf42, Arap3, Elovl7, Rapgef6, Phf3, Maml3, Mtx3, Gabra2, Hdgfl1, Epha5, Rnf4, Etfdh, C2orf15, Ttc39b, Pcbd2, Tma16, Myo10, Rfesd, Ctc-432m15.3, Bmpr1b, Dag1, Rnf150, Gabrp, Clvs1, Penk, Hdac2, Heph, C8orf44, Caap1, Ccnc, Brf2, Pik3r1, Tram1, Smim12, Nudt18, Fam196a, Kcnb2, Kif13b, Maml2, Slc35f2, Cd44, Mogat2, Traf6, Hinfp, Grik4, Rnf141, Gramd1b, Sptbn2, Fbxo3, Gmds, Rbm4b, Rgs4, Plekha7, Fzd4, C4orf50, Gpr68, Ms4a14, C11orf57, Sergef, Tmem107, Rab30, Prss23, Sipa1, Ncapd3, Calu, C5orf63, Lrba, Ddah1, Kiaa1147, Sp7, Muc12, Far2, C21orf62, Cdk8, Dopey1, Rergl, Pdzd9, Pabpc1l, Tinagl1, Asb11, Col14a1, Kiaa0319, Bcl7a, Pla2g7, Abcc4, Slamf1, Sox15, Cep57, Fam133a, Dcaf12l2, Zfx, Oat, Gls2, Smap2, Cd300lg, Znf362, Atp1b3, Mccc1, Tfrc, Nhsl2, Mphosph9, Psme3, Scarb1, Celf5, Acat2, Pdcd2, Ptbp2, Habp2, Trdn, Nup93, C16orf52, Dnd1, Itih1, Pcdh7, Lrtm2, C19orf55, Chd4, Rnd2, Abca13, C1rl, Smg6, C12orf66, Coro1b, Sphk1, Ak4, Tet2, Stxbp5, Sox5, Armc6, Egln3, Cntn1, Ash1l, Chst11, Krt74, Ppfia2, Tmem19, Atxn2, Frs2, Rp11-644f5.10, Acvrl1, Tmprss12, Myl6, Prr24, Spats2, Gtf2a1, Dnal1, Hectd1, Ly75, Akt1, Vti1b, Map3k9, C14orf132, Yaf2, Pgf, Prox2, Ift43, Acin1, Dhrs4, Shisa9, Tle3, Fam63b, Meis1, Synm, Idh3a, Znf423, Rp11-315d16.2, Szt2, Cdip1, Def8, Rp6-24a23.6, Dnase1l2, Myo9a, Stoml1, Apobr, Sall1, Snx29, Ct62, Tbc1d24, Slc6a2, Cdyl2, Ppp1r27, Scarf1, Asgr1, Nup88, Card14, C17orf74, Nxn, Atpaf1, Smim6, Pirt, Ccl2, Tmem200c, Twsg1, Cd226, Fam222b, Cbln2, Rasgrp4, Ac139100.2, Fbf1, Nle1, Slc16a2, Tmem101, Hspb6, Znhit3, Ramp2, Lrfn3, Tmc6, Znf227, Fosb, Syt5, Aoc3, Mex3c, Dyrk1b, Ac008964.1, Zfp36, Al031663.2, Znf814, Tead2, Pak4, Lmtk3, Ac010441.1, Znf558, Ap003733.1, Eps15l1, Tp53aip1, Dnajc3, Macf1, Il11ra, Ptp4a2, Ac007040.11, Tbx18, Cnksr3, Exoc7, Slc25a16	2177

Among these candidates, FOXO3 emerged as a particularly compelling target. Gene Ontology analysis revealed that FOXO3 is strongly associated with transcriptional regulation, and KEGG pathway analysis showed enrichment in the AMPK signaling pathway and Chemokine signaling pathway, both of which are closely related to CXCR5-mediated signaling (Fig. 6G**, **Table S5-9). To determine whether FOXO3 serves as a critical intermediate target, we first individually overexpressed three candidate microRNAs in vitro. The results showed that overexpression of all three microRNAs led to varying degrees of downregulation of FOXO3 protein expression (Fig. 6H), suggesting that FOXO3 may be negatively regulated by these microRNAs. However, changes in expression alone are insufficient to establish a direct targeting relationship. Therefore, we further constructed a luciferase reporter vector containing the FOXO3 3′ untranslated region (3′UTR) to investigate whether these microRNAs could directly bind to the FOXO3 3′UTR and suppress its post-transcriptional expression. Overexpression of miR-214-5p significantly suppressed FOXO3 3′UTR luciferase activity, while miR-23b-5p and miR-6239 also exerted inhibitory effects to varying degrees (Fig. 6I), demonstrating that FOXO3 is a direct target of these microRNAs. Consistently, in vivo analysis showed that *Foxo3* mRNA levels were significantly reduced in the Sp5C of model mice, whereas EA treatment markedly reversed this reduction, in agreement with the in vitro findings (Fig. 6J). Further prediction of microRNA binding sites within the FOXO3 3′UTR revealed that miR-6239 harbors two binding sites (Site-1: 793–799; Site-2: 1127–1133), while miR-214-5p (Site-3: 2270–2276) and miR-23b-5p (Site-4: 2773–2779) each possess a single binding site. These results support a model in which FOXO3 is cooperatively suppressed by multiple microRNAs (Fig. S3B).

Collectively, these findings indicate that EA downregulates miR-23b-5p, miR-214-5p, and miR-6239, thereby relieving their inhibitory effects on FOXO3 and restoring FOXO3-mediated transcriptional repression of CXCR5. This defines a previously unrecognized microRNA–FOXO3–CXCR5 regulatory axis underlying EA-mediated neuromodulation.

## Discussion

Growing evidence implicates neuroinflammation as a critical driver of migraine pathogenesis and chronification [36], with chemokine signaling playing a central role in this process [37]. Although several chemokine axes, such as CXCL1/CXCR2 and CCL2/CCR2, have been well documented in migraine, most existing studies have focused on their canonical and extensively characterized signaling mechanisms [38]. In the present study, we aimed to explore whether a distinct and previously underappreciated chemokine signaling pathway might also participate in migraine pathophysiology. In contrast, the CXCL13/CXCR5 axis is characterized by a high degree of molecular specificity, with CXCL13 being the principal ligand for CXCR5. This relatively exclusive pairing provides a more precise framework for dissecting neuroimmune interactions, minimizing the functional redundancy commonly observed among other chemokine systems. Evidence from pain models has suggested that hyperexcitable neurons can release CXCL13, which selectively targets CXCR5 expressed on astrocytes [39]. These astrocyte-derived factors, in turn, enhance neuronal synaptic excitability, forming a feed-forward loop that sustains pain hypersensitivity. Besides, he CXCL13/CXCR5 axis has been characterized in various inflammatory conditions including aging [40], cancer [41, 42], and autoimmune diseases [43–45]. Our data also demonstrated that migraine pathology involves CXCL13/CXCR5-mediated activation of ERK signaling [46] and subsequent release of pro-inflammatory cytokines (IL-6, CCL2) and neuropeptides (SP, PACAP). Notably, recent clinical studies have re-emphasized SP as an important mediator in migraine, reinforcing its relevance to trigeminovascular activation and meningeal vasodilation [47, 48]. In parallel, PACAP has been increasingly implicated not only in migraine but also in other primary headache disorders, highlighting a broader role for PACAP-driven neurogenic inflammation across headache pathophysiology [49]. Together, these findings position the CXCL13/CXCR5–ERK axis as a convergent upstream mechanism regulating neuropeptide release and suggest its potential relevance beyond migraine to related headache conditions. EA treatment effectively normalized these pathological changes, attenuating both nociceptive behaviors and molecular markers of neuroinflammation [24, 50, 51]. The therapeutic specificity of EA was further established through gain- and loss-of-function experiments [51]. In the present study, we observed that CXCR5 overexpression aggravated migraine-like behaviors and neurochemical disturbances, whereas CXCR5 knockdown produced effects comparable to those induced by EA intervention. These findings not only underscore the pivotal role of CXCR5 in migraine pathophysiology but also suggest that EA may exert its analgesic and anti-inflammatory effects through modulation of CXCR5-related signaling.

Importantly, microRNA sequencing further revealed a set of differentially expressed microRNAs following EA treatment, providing novel insight into the upstream regulatory landscape of the CXCL13/CXCR5 axis. Functional validation indicated that these microRNAs act as intermediate regulators by convergently targeting FOXO3, thereby influencing CXCR5-driven ERK signaling and downstream neuronal excitability. Collectively, these observations support a mechanistic model in which EA modulates a “microRNA–FOXO3–CXCR5–ERK” regulatory cascade to attenuate migraine-associated nociceptive sensitization.

**Fig. 7:**
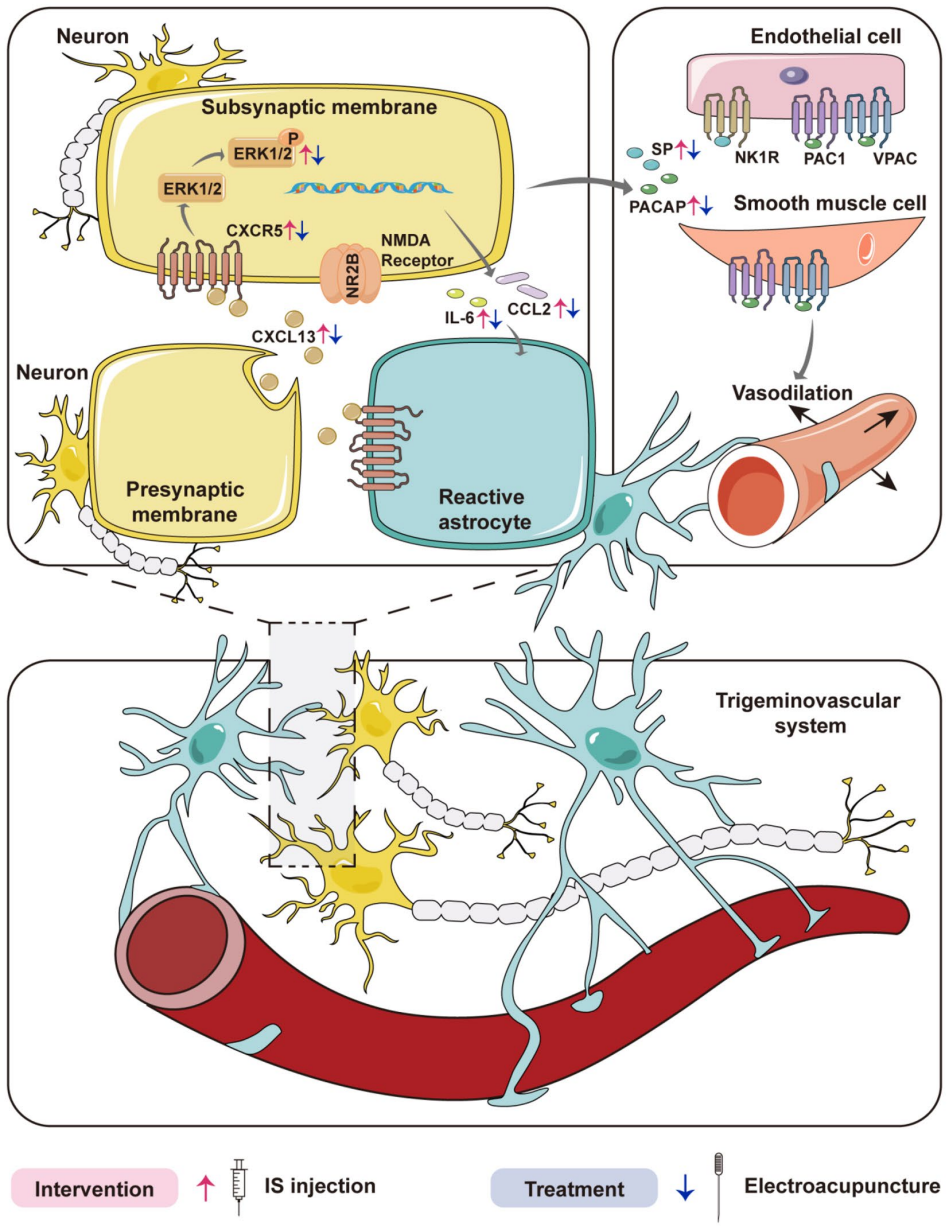
Proposed mechanisms of acupuncture in alleviating neuroinflammation and pain in migraine. Red arrows indicate IS-induced up-regulation of certain molecules. Green arrows indicating EA-induced down regulation of certain molecules. IS: inflammatory soup; EA: electroacupuncture

The CXCL13/CXCR5 axis has been implicated in pain modulation through multiple downstream signaling pathways, including ERK and NF-κB, as demonstrated in previous studies [52, 53]. While this chemokine system has been investigated in various cancer-related pain conditions such as pancreatic cancer, breast cancer, and bone cancer [41, 54, 55], as well as in immune regulation through its effects on T cell and B cell responses [43, 56],our study provides novel evidence for its involvement in migraine pathophysiology. We observed significant ERK phosphorylation in the Sp5C of migraine model mice, which was effectively normalized by electroacupuncture treatment. These findings extend previous reports of peripheral inflammatory markers in migraine models [24] by demonstrating specific central neuroinflammatory changes in key pain-processing regions. Our results reveal that migraine-associated neuroinflammation involves elevated IL-6 and CCL2 expression in the Sp5C, consistent with clinical observations of central sensitization in migraine patients [57]. The electroacupuncture-mediated reduction of these inflammatory markers, along with decreased ERK activation, suggests a specific mechanism of action through the CXCL13/CXCR5/ERK pathway. This interpretation is supported by established knowledge that IL-6 serves as a downstream effector of ERK signaling [35, 58], and that EA can regulate downstream inflammatory signals through the CXCL13/CXCR5/ERK pathway (Fig. 7). These findings provide a mechanistic basis for EA's clinical efficacy in migraine treatment while identifying CXCL13/CXCR5 signaling as a potential new target for migraine therapy. Future studies should explore whether these mechanisms are conserved across different migraine subtypes and in human patients.

Growing evidence highlights the importance of neuroinflammation and glial-neuronal interactions in migraine pathophysiology, with our findings demonstrating significant activation of both neurons and astrocytes in the Sp5C region of migraine model mice [59]. However, despite the presence of astrocytic activation, cellular localization analyses demonstrated that CXCL13 was primarily colocalized with neurons, while CXCR5 exhibited predominant neuronal expression with only minor astrocytic presence. This neuron-enriched expression pattern indicates that CXCL13/CXCR5 signaling in the Sp5C is unlikely to function mainly as a glia-mediated bridge between astrocyte activation and neuronal hyperexcitability. Instead, these findings support a model in which the CXCL13/CXCR5 axis predominantly facilitates direct neuron-to-neuron communication within the trigeminovascular system. Based on the neuronal co-localization of CXCL13 and CXCR5, we propose a working model in which CXCL13 released from primary sensory neurons could potentially act on CXCR5 expressed by neighboring neurons, thereby influencing neuronal excitability and synaptic transmission. However, this interpretation remains speculative, as immunofluorescence co-localization alone does not establish functional neuron-to-neuron communication. Definitive validation of direct neuronal signaling will require higher-resolution approaches, such as electron microscopy or other ultrastructural and functional techniques, to determine the precise subcellular localization of CXCL13 and CXCR5 and to assess their involvement at synaptic or perisynaptic sites.

Several limitations should be acknowledged. First, the cellular distribution of phosphorylated ERK in migraine conditions requires further investigation to determine the relative contributions of neuronal versus astrocytic CXCR5 signaling. Second, while ERK represents an important downstream target, additional pathways such as NF-κB [52, 60] may contribute to EA's effects and warrant exploration using specific pathway inhibitors. Third, the incomplete reversal of allodynia following CXCR5 silencing suggests involvement of additional pain mechanisms beyond the CXCL13/CXCR5 axis and further work using pathway-specific inhibitors or genetic approaches is required to dissect these parallel pathways. Fourth, while sham EA was applied at non-acupoint locations to control for acupoint specificity, identical electrical stimulation was still delivered, which may itself exert neuromodulatory effects independent of acupoint activation. Therefore, future studies should incorporate an additional control group receiving GB20 and GB34 acupuncture without electrical stimulation to more precisely distinguish acupoint-specific effects from those attributable to electrical stimulation alone. Fifth, the potential contributions of immune cells (T cells, macrophages) to migraine-related neuroinflammation [61] were not addressed in this study. Finally, this study was conducted exclusively in male mice to minimize variability associated with the estrous cycle and to ensure robust mechanistic resolution. However, given the well-established sex differences in migraine prevalence and the modulatory effects of sex hormones on trigeminovascular activity, the exclusion of female subjects limits the immediate translational relevance of our findings [2].

Clinical neuroimaging studies have identified the Sp5C as a key node in migraine pathophysiology, with acupuncture shown to normalize Sp5C-centered functional abnormalities [62]. These observations parallel our preclinical findings that CXCR5 signaling within the Sp5C regulates ERK activation and downstream neuroinflammatory responses, contributing to the anti-inflammatory and analgesic effects of EA in a migraine model. Notwithstanding these insights, our data remain hypothesis-generating and cannot be directly extrapolated to patients. The relevance of the CXCL13/CXCR5/ERK pathway to human migraine and to the clinical effects of EA requires validation in human tissues or trigeminal pathways. Future studies integrating CXCL13/CXCR5-related molecular profiling with neuroimaging measures of Sp5C function may strengthen translational relevance and inform the development of targeted therapeutic strategies. Importantly, such efforts are further motivated by clinical evidence indicating that currently available prophylactic treatments for migraine are frequently limited by modest efficacy, poor long-term adherence, and treatment-limiting adverse effects, underscoring a clear unmet need for mechanism-driven and better-tolerated therapeutic approaches.

## Conclusion

Our study demonstrates that neuroinflammatory mechanisms mediated through the CXCL13/CXCR5/ERK signaling axis contribute significantly to migraine pathogenesis. With FOXO3 acting as an intermediate regulatory target, EA may restore FOXO3 expression to suppress CXCR5 transcription, thereby exerting potent anti-nociceptive and anti-inflammatory effects by selectively modulating this pathway and effectively attenuating both pain sensitization and neuroinflammatory responses in migraine models. The robust therapeutic outcomes observed in our preclinical study, coupled with existing clinical evidence of EA's efficacy in migraine management, strongly support CXCR5 as a promising molecular target for migraine therapy. Future translational research should focus on validating these findings in human patients and exploring the development of CXCR5-targeted interventions, which may offer novel treatment strategies for migraine, particularly for cases refractory to conventional therapies [63, 64].

## Supplementary Information

Supplementary Material 1.Fig. S1 Repeated dural injection of IS in C57/BL6J mice. **A** Injection sites for IS/PBS. **B** Acupuncture points used for electroacupuncture intervention. **C** Flowchart of Experiment 1 design. IS: inflammatory soup

Supplementary Material 2.Fig. S2 Microinjection of AAV-*Cxcr5* in Sp5C region in C57/BL6J mice. **A**, **B** Microinjection sites for virus. **C** Flowchart of Experiment 2 design

Supplementary Material 3. Fig. S3 Validation of microRNA expression and predicted binding sites on FOXO3. **A** Quantitative PCR analysis showing relative expression levels of miR-12179-5p, miR-23b-5p, miR-214-5p, and miR-6239 in Neuro-2a cells following transfection with the corresponding microRNA mimics or negative controlmimics. Data are presented as mean ± SD. **B** Schematic representation of predicted binding sites for miR-6239, miR-214-5p, and miR-23b-5p within the 3′ untranslated regionof the mouse FOXO3 transcript, as identified by bioinformatic prediction. The positions of individual binding sites and their corresponding nucleotide coordinates are indicated. Data are presented as mean ± SD. *P < 0.05, **P < 0.01, ***P < 0.001, ****P < 0.0001; ns, not significant

Supplementary Material 4

Supplementary Material 5

Supplementary Material 6

Supplementary Material 7

Supplementary Material 8

Supplementary Material 9

Supplementary Material 10

Supplementary Material 11

Supplementary Material 12

## Data Availability

The data used in this article will be publicly available at the Gene Expression Omnibus (https://www.ncbi.nlm.nih.gov/geo/), under accession number GSE281596.

## Abbreviations

AAV: Adenovirus-associated virus
CM: Chronic migraine
CNS: Central nervous system
EA: Electroacupuncture
ELISA: Enzyme-linked immunosorbent assay
IS: Inflammatory soup
IF: Immunofluorescence
NC: Negative control
PNS: Peripheral nervous system
SEA: Sham electroacupuncture
WB: Western blotting
