# Supplementary figures and images for: Electroacupuncture alleviates migraine through CXCL13/CXCR5-mediated communication
Source: Chin Med. 2026 Feb 2;21:59. doi: 10.1186/s13020-026-01338-8 (PMC12866310; doi:10.1186/s13020-026-01338-8)

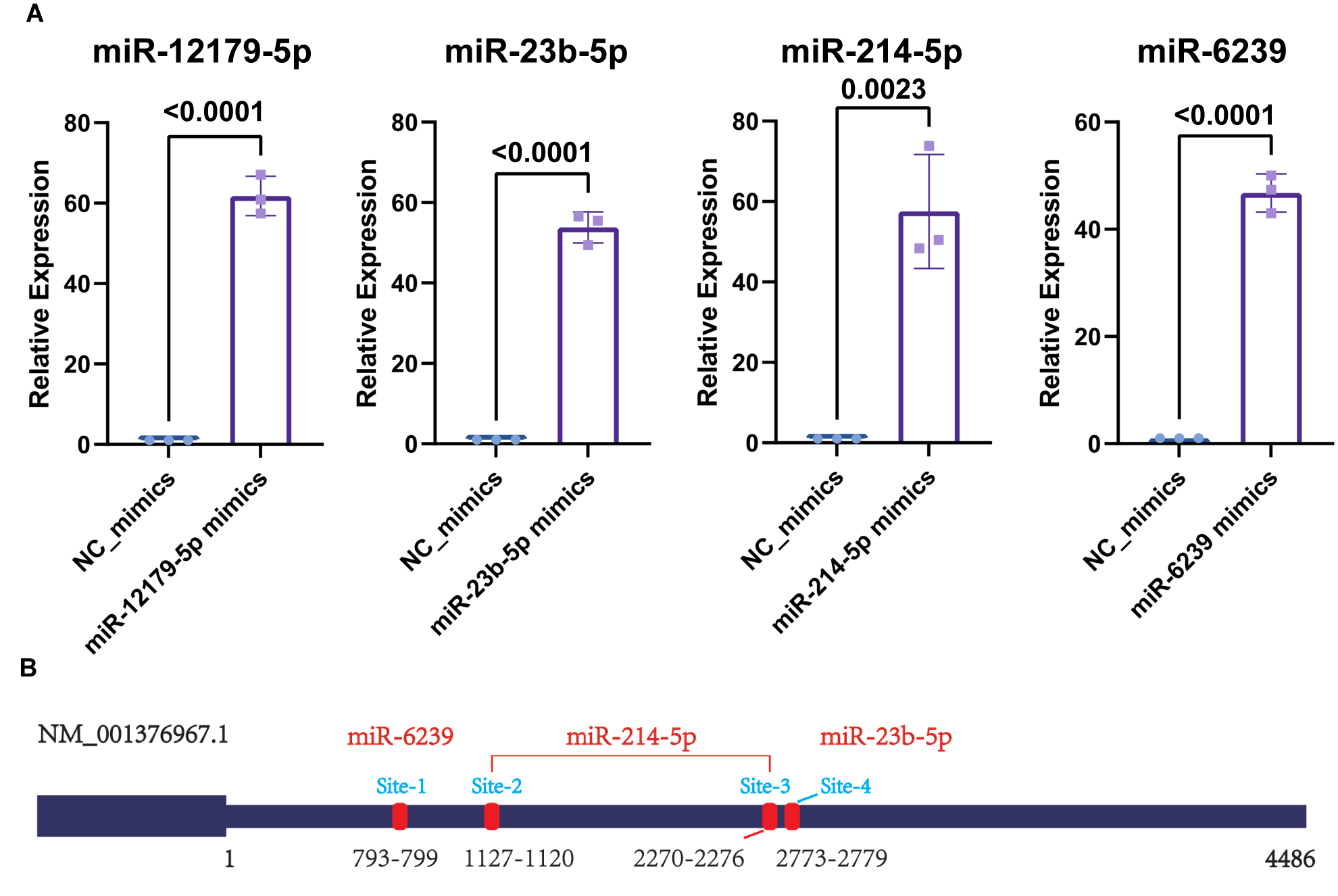

Supplement: Supplementary file 3 — Supplementary Material 3. Fig. S3 Validation of microRNA expression and predicted binding sites on FOXO3. A Quantitative PCR analysis showing relative expression levels of miR-12179-5p, miR-23b-5p, miR-214-5p, and miR-6239 in Neuro-2a cells following transfection with the corresponding microRNA mimics or negative controlmimics. Data are presented as mean ± SD. B Schematic representation of predicted binding sites for miR-6239, miR-214-5p, and miR-23b-5p within the 3′ untranslated regionof the mouse FOXO3 transcript, as identified by bioinformatic prediction. The positions of individual binding sites and their corresponding nucleotide coordinates are indicated. Data are presented as mean ± SD. *P < 0.05, **P < 0.01, ***P < 0.001, ****P < 0.0001; ns, not significant [file 13020_2026_1338_MOESM3_ESM.tif]
