# Supplementary material for: Electroacupuncture alleviates migraine through CXCL13/CXCR5-mediated communication
Source: Chin Med. 2026 Feb 2;21:59. doi: 10.1186/s13020-026-01338-8 (PMC12866310; doi:10.1186/s13020-026-01338-8)
Supplement: Supplementary file 4 — Supplementary Material 4 [file 13020_2026_1338_MOESM4_ESM.docx]

**Table S1. Antibodies used in western blotting and immunofuorescence analysis**

| Antibody | Manufacturer | Applied ratio | Catalog number | Host |
| --- | --- | --- | --- | --- |
| For immunofuorescence analysis | | | | |
| CXCL13 | Abcam | 1:500 | Ab199043 | Rabbit |
| CXCR5 Rabbit mAb | Abclonal | 1:1000 | A8950 | Rabbit |
| GFAP | Servicebio | 1:1000 | GB11096 | Rabbit |
| Iba-1 | CST | 1:100 | 17198S | Rabbit |
| NeuN | Servicebio | 1:2000 | GB11138 | Rabbit |
| Anti-Rabbit lgG (HRP) | Servicebio | 1:800 | GB21303 | Rabbit |
| For Western blotting analysis | | | | |
| CXCL13 | Abclonal | 1:500 | A15782 | Rabbit |
| CXCR5 Rabbit mAb | Abclonal | 1:1000 | A8950 | Rabbit |
| p44/42 MAPK (Erk1/2) | CST | 1:5000 | 4695S | Raabit |
| Phospho-p44/42 MAPK (Erk1/2) | CST | 1:500 | 4370S | Rabbit |
| FOXO3 | Proteintech | 1:2000 | 10849-1-AP | Rabbit |
| β-actin | Servicebio | 1:1000 | GB11001 | Rabbit |
| Anti-Rabbit lgG (HRP) | Servicebio | 1:3000 | GB23303 | Rabbit |

CXCL13: C-X-C motif chemokine ligand 13；CXCR5: C-X-C chemokine receptor type 5；mAb: Monoclonal antibody；GFAP: Glial fibrillary acidic protein；Iba-1: Ionized calcium-binding adapter molecule 1；NeuN: Neuronal nuclei；IgG: Immunoglobulin G；HRP: Horseradish peroxidase；p44/42 MAPK: p44/42 mitogen-activated protein kinase (Erk1/2)；Phospho-p44/42 MAPK: Phosphorylated p44/42 mitogen-activated protein kinase (Erk1/2)；FOXO3: Forkhead box O3; β-actin: Beta-actin
