# Supplementary material for: Electroacupuncture alleviates migraine through CXCL13/CXCR5-mediated communication
Source: Chin Med. 2026 Feb 2;21:59. doi: 10.1186/s13020-026-01338-8 (PMC12866310; doi:10.1186/s13020-026-01338-8)
Supplement: Supplementary file 5 — Supplementary Material 5 [file 13020_2026_1338_MOESM5_ESM.docx]

**Table S2. Primer sequences used for qPCR analysis.**

| **Gene** | **Derection** | **Primer (5’-3’)** |
| --- | --- | --- |
| IL-6 | Forward(F) | CCCCAATTTCCAATGCTCTCC |
|  | Reverse(R) | CGCACTAGGTTTGCCGAGTA |
| CCL2 | F | CAGGTCCCTGTCATGCTTCT |
|  | R | GTGGGGCGTTAACTGCATCT |
| GAPDH | F | CCTCGTCCCGTAGACAAAATG |
|  | R | TGAGGTCAATGAAGGGGTCGT |
| CXCR5 | F | CTGTTCGCCTTACCGGAACT |
|  | R | GCATCGGTAGTAGGAAGCCC |
| FOXO3 | F | CGTCTCTGAACTCCTTGCGT |
|  | R | TCTGGTTGCCGTAGTGTGAC |

IL-6: Interleukin 6；CCL2: C-C Motif Chemokine ligand 2；GAPDH: Glyceraldehyde 3-phosphate dehydrogenase; CXCR5: C-X-C chemokine receptor type 5; FOXO3: Forkhead box O3
