# Supplementary material for: Electroacupuncture alleviates migraine through CXCL13/CXCR5-mediated communication
Source: Chin Med. 2026 Feb 2;21:59. doi: 10.1186/s13020-026-01338-8 (PMC12866310; doi:10.1186/s13020-026-01338-8)
Supplement: Supplementary file 6 — Supplementary Material 6 [file 13020_2026_1338_MOESM6_ESM.docx]

**Table S3.** **Behavioral testing outcome measure in Experimental Design 1.**

| **Outcome Measure** | | | | | **Pairwise Comparison** | | | | | |
| --- | --- | --- | --- | --- | --- | --- | --- | --- | --- | --- |
|  |  |  |  |  | **IS vs. Con** | | **IS vs. IS+EA** | | **IS vs. IS+SEA** | |
| **Difference from Baseline (Day 0) in Frequency of IS dural injection, Mean (SE)** | | | | | | | | | | |
| **50% Face Mechanical Withdrawal Threshold (g) (Ipsilateral)** | | | | | | | | | | |
|  | **Con**  **(n=12)** | **IS**  **(n=12)** | **IS+EA (n=12)** | **IS+SEA (n=12)** | **Effect Size (95% CI)** | ***P* Value** | **Effect Size (95% CI)** | ***P* Value** | **Effect Size (95% CI)** | ***P* Value** |
| **Day 0** | 0.28(0.04) | 0.30(0.04) | 0.32(0.03) | 0.30(0.05) | 0.02  (-0.08 to 0.12) | 0.94 | 0.02  (-0.12 to 0.083) | 0.94 | 0.003  (-0.12 to 0.12) | >0.99 |
| **Day 1** | 0.34(0.03) | 0.26(0.04) | 0.32(0.03) | 0.26(0.05) | -0.080  (-0.14 to 0.02) | 0.17 | -0.06  (-0.16 to 0.04) | 0.39 | -0.003  (-0.12 to 0.12) | >0.99 |
| **Day 3** | 0.34(0.04) | 0.13(0.01) | 0.29(0.04) | 0.13(0.02) | -0.2  (-0.31 to -0.10) | <0.0001 | -0.16  (-0.27 to -0.06) | 0.0007 | 0  (-0.13 to 0.13) | >0.99 |
| **Day 5** | 0.36(0.03) | 0.09(0.01) | 0.25(0.04) | 0.12(0.02) | -0.27  (-0.37 to -0.16) | <0.0001 | -0.160  (-0.26 to -0.06) | 0.0009 | -0.02  (-0.15 to 0.10) | 0.9558 |
| **Day 7** | 0.32(0.03) | 0.07(0.01) | 0.26(0.04) | 0.07(0.01) | -0.25  (-0.35 to -0.15) | <0.0001 | -0.19  (-0.29 to -0.09) | <0.0001 | 0.006  (-0.12 to 0.13) | 0.9991 |
| **Day 8** | 0.30(0.04) | 0.06(0.01) | 0.28(0.04) | 0.06(0.02) | -0.24  (-0.34 to -0.14) | <0.0001 | -0.22  (-0.32 to -0.12) | <0.0001 | 5.551E-17  (-0.13 to 0.13) | >0.99 |
| **50% Face Mechanical Withdrawal Threshold (g) (Contralateral)** | | | | | | | | | | |
|  | **Con**  **(n=12)** | **IS**  **(n=12)** | **IS+EA (n=12)** | **IS+SEA (n=12)** | **Effect Size (95% CI)** | ***P* Value** | **Effect Size (95% CI)** | ***P* Value** | **Effect Size (95% CI)** | ***P* Value** |
| **Day 0** | 0.30(0.04) | 0.30(0.04) | 0.32(0.03) | 0.33(0.04) | 0  (-0.11 to 0.11) | >0.99 | -0.02  (-0.13 to 0.09) | 0.95 | -0.03  (-0.16 to 0.10) | 0.89 |
| **Day 1** | 0.32(0.03) | 0.24(0.03) | 0.28(0.04) | 0.23(0.04) | -0.08  (-0.19 to 0.03) | 0.21 | -0.04  (-0.15 to 0.07) | 0.78 | 0.01  (-0.12to 0.14) | 0.99 |
| **Day 3** | 0.28(0.04) | 0.14(0.01) | 0.26(0.04) | 0.13(0.02) | -0.14  (-0.25 to -0.03) | 0.0083 | -0.12  (-0.23 to -0.01) | 0.0240 | 0.01  (-0.13 to 0.14) | 0.9985 |
| **Day 5** | 0.30(0.04) | 0.09(0.01) | 0.26(0.04) | 0.10(0.02) | -0.21  (-0.32 to -0.10) | <0.0001 | -0.17  (-0.28 to -0.06) | 0.0011 | -0.01  (-0.14 to 0.13) | 0.9985 |
| **Day 7** | 0.30(0.04) | 0.06(0.01) | 0.28(0.04) | 0.07(0.01) | -0.23  (-0.34 to -0.12) | <0.0001 | -0.22  (-0.33 to -0.11) | <0.0001 | -0.002  (-0.14 to 0.13) | >0.9999 |
| **Day 8** | 0.28(0.04) | 0.06(0.01) | 0.28(0.04) | 0.08(0.02) | -0.22  (-0.33 to -0.11) | <0.0001 | -0.22  (-0.33 to -0.11) | <0.0001 | -0.02  (-0.15 to 0.12) | 0.9816 |
| **50% Hindpaw Mechanical Withdrawal Threshold (g) (Ipsilateral)** | | | | | | | | | | |
|  | **Con**  **(n=12)** | **IS**  **(n=12)** | **IS+EA (n=12)** | **IS+SEA (n=12)** | **Effect Size (95% CI)** | ***P* Value** | **Effect Size (95% CI)** | ***P* Value** | **Effect Size (95% CI)** | ***P* Value** |
| **Day 0** | 1.31(0.14) | 1.23(0.08) | 1.31(0.14) | 1.37(0.13) | -0.09  (-0.45 to 0.27) | 0.8962 | -0.09  (-0.45 to 0.27) | 0.8962 | -0.14  (-0.50 to 0.22) | 0.665 |
| **Day 1** | 1.37(0.13) | 1.06(0.10) | 1.37(0.13) | 1.11(0.07) | -0.31  (-0.67 to 0.04) | 0.1007 | -0.31  (-0.67 to 0.05) | 0.1007 | -0.06  (-0.42 to 0.30) | 0.9651 |
| **Day 3** | 1.23(0.08) | 0.69(0.09) | 1.26(0.14) | 0.74(0.09) | -0.54  (-0.90 to -0.18) | 0.0014 | -0.57  (-0.93 to -0.21) | 0.0007 | -0.06  (-0.42 to 0.30) | 0.9651 |
| **Day 5** | 1.23(0.08) | 0.39(0.05) | 1.20(0.17) | 0.42(0.07) | -0.83  (-1.19 to -0.47) | <0.0001 | -0.81  (-1.16 to -0.45) | <0.0001 | -0.02  (-0.38 to 0.34) | 0.9976 |
| **Day 7** | 1.31(0.14) | 0.23(0.04) | 1.00(0.12) | 0.26(0.05) | -1.01  (-1.45 to -0.73) | <0.0001 | -0.77  (-1.13 to -0.41) | <0.0001 | -0.03  (-0.39 to 0.33) | 0.992 |
| **Day 8** | 1.34(0.18) | 0.21(0.05) | 1.11(0.07) | 0.23(0.04) | -1.13  (-1.49 to -0.77) | <0.0001 | -0.90  (-1.26 to -0.54) | <0.0001 | -0.02  (-0.38 to 0.34) | 0.999 |
| **50% Hindpaw Mechanical Withdrawal Threshold (g) (Contralateral)** | | | | | | | | | | |
|  | **Con**  **(n=12)** | **IS**  **(n=12)** | **IS+EA (n=12)** | **IS+SEA (n=12)** | **Effect Size (95% CI)** | ***P* Value** | **Effect Size (95% CI)** | ***P* Value** | **Effect Size (95% CI)** | ***P* Value** |
| **Day 0** | 1.46(0.16) | 1.54(0.17) | 1.40(0.17) | 1.74(0.12) | -0.09  (-0.31 to 0.49) | 0.9210 | 0.14  (-0.26 to 0.54) | 0.7298 | -0.20  (-0.60 to 0.20) | 0.4952 |
| **Day 1** | 1.37(0.13) | 1.06(0.10) | 1.51(0.14) | 1.06(0.06) | -0.31  (-0.71 to 0.09) | 0.1563 | -0.46  (-0.86 to -0.06) | 0.0202 | 0  (-0.40 to 0.40) | >0.9999 |
| **Day 3** | 1.37(0.13) | 0.74(0.09) | 1.26(0.14) | 0.63(0.07) | -0.63  (-1.0 to -0.23) | 0.0008 | -0.51  (-0.91 to -0.12) | 0.0075 | 0.11  (-0.29 to 0.51) | 0.8368 |
| **Day 5** | 1.37(0.13) | 0.30(0.05) | 1.20(0.17) | 0.33(0.06) | -1.07  (-1.47 to -0.68) | <0.0001 | -0.90  (-1.30 to -0.50) | <0.0001 | -0.03  (-0.43 to 0.37) | 0.9966 |
| **Day 7** | 1.40(0.17) | 0.18(0.04) | 1.11(0.11) | 0.23(0.04) | -1.22  (-1.62 to -0.82) | <0.0001 | -0.93  (-1.33 to -0.53) | <0.0001 | -0.05  (-0.45 to 0.35) | 0.9850 |
| **Day 8** | 1.49(0.19) | 0.23(0.04) | 1.17(0.08) | 0.26(0.05) | -1.26  (-1.66 to -0.86) | <0.0001 | -0.94  (-1.34 to -0.54) | <0.0001 | -0.03  (-0.43 to 0.37) | 0.9941 |
| **Tail Flick Latency (s)** | | | | | | | | | | |
|  | **Con**  **(n=12)** | **IS**  **(n=12)** | **IS+EA (n=12)** | **IS+SEA (n=12)** | **Effect Size (95% CI)** | ***P* Value** | **Effect Size (95% CI)** | ***P* Value** | **Effect Size (95% CI)** | ***P* Value** |
| **Day 0** | 3.56(0.10) | 3.62(0.09) | 3.59(0.08) | 3.50(0.33) | 0.06  (-0.29 to 0.41) | 0.9381 | 0.03  (-0.28 to 0.35) | 0.9778 | 0.12  (-0.89 to 1.13) | 0.9666 |
| **Day 1** | 3.55(0.11) | 2.94(0.20) | 3.53(0.07) | 3.14(0.09) | -0.61  (-1.2 to 0.026) | 0.0600 | -0.59  (-1.21 to 0.04) | 0.0638 | -0.20  (-0.82 to 0.43) | 0.7060 |
| **Day 3** | 3.60(0.12) | 2.54(0.12) | 3.46(0.05) | 2.79(0.12) | -1.06  (-1.53 to -0.59) | 0.0002 | -0.92  (-1.30 to -0.54) | 0.0004 | -0.25  (-0.70 to 0.20) | 0.3528 |
| **Day 5** | 3.51(0.08) | 1.78(0.10) | 3.08(0.09) | 2.03(0.09) | -1.72  (-2.08 to -1.37) | <0.0001 | -1.29  (-1.66 to -0.92) | <0.0001 | -0.24  (-0.62 to 0.13) | 0.2358 |
| **Day 7** | 3.45(0.07) | 1.55(0.09) | 3.02(0.10) | 1.84(0.11) | -1.90  (-2.20 to -1.61) | <0.0001 | -1.47  (-1.83 to -1.11) | <0.0001 | -0.29  (-0.66 to 0.09) | 0.1420 |
| **Day 8** | 3.46(0.10) | 2.31(0.13) | 3.32(0.02) | 2.30(0.13) | -1.16  (-1.60 to -0.71) | <0.0001 | -1.01  (-1.41 to -0.62) | 0.0005 | -0.01  (-0.47 to 0.50) | 0.9999 |
| **Hot-plate Latency (s)** | | | | | | | | | | |
|  | **Con**  **(n=12)** | **IS**  **(n=12)** | **IS+EA (n=12)** | **IS+SEA (n=12)** | **Effect Size (95% CI)** | ***P* Value** | **Effect Size (95% CI)** | ***P* Value** | **Effect Size (95% CI)** | ***P* Value** |
| **Day 0** | 13.57(1.42) | 13.06(1.13) | 13.90(0.48) | 14.60(0.71) | -0.50  (-3.06 to 2.05) | 0.9369 | -0.84  (-3.50 to 1.82) | 0.7960 | -1.54  (-4.09 to 1.01) | 0.3443 |
| **Day 1** | 15.51(0.84) | 12.38(0.74) | 14.04(0.72) | 12.96(0.80) | -3.14  (-5.69 to -0.58) | 0.0114 | -1.66  (-4.32 to 0.99) | 0.3189 | -0.58  (-3.24 to 2.07) | 0.9174 |
| **Day 3** | 16.05(0.59) | 8.48(0.33) | 12.94(0.84) | 10.07(0.76) | -7.56  (-10.45 to -4.68) | <0.0001 | -4.46  (-7.21 to -1.71) | 0.0006 | -1.59  (-4.24 to 1.06) | 0.3502 |
| **Day 5** | 15.01(0.74) | 4.71(0.31) | 11.84(0.74) | 7.35(0.81) | -10.30  (-12.85 to -7.75) | <0.0001 | -7.13  (-9.78 to -4.47) | <0.0001 | -2.64  (-5.30 to 0.01) | 0.0515 |
| **Day 7** | 14.54(0.56) | 3.93(0.56) | 11.82(1.01) | 6.23(0.66) | -10.61  (-13.16 to -8.06) | <0.0001 | -7.89  (-10.54 to -5.23) | <0.0001 | -2.30  (-4.96 to 0.36) | 0.1060 |
| **Day 8** | 15.71(0.55) | 3.98(0.94) | 11.18(0.65) | 5.80(1.09) | -11.73  (-14.38 to -9.07) | <0.0001 | -7.20  (-10.09 to -4.31) | <0.0001 | -1.82  (-4.70 to 1.08) | 0.3155 |
