# Supplementary material for: Electroacupuncture alleviates migraine through CXCL13/CXCR5-mediated communication
Source: Chin Med. 2026 Feb 2;21:59. doi: 10.1186/s13020-026-01338-8 (PMC12866310; doi:10.1186/s13020-026-01338-8)
Supplement: Supplementary file 7 — Supplementary Material 7 [file 13020_2026_1338_MOESM7_ESM.docx]

**Table S4.** **Behavioral testing outcome measure in Experimental Design 2.**

|  | | | | | | | **Pairwise Comparison** | | | | | |
| --- | --- | --- | --- | --- | --- | --- | --- | --- | --- | --- | --- | --- |
| **Outcome Measure** | | | | | | | **IS vs AAV-Cxcr5+IS** | | **IS vs ShRNA+IS** | | **AAV-Cxcr5+IS vs**  **AAV-Cxcr5+IS+EA** | |
| **Difference from Baseline (Day 0) in Frequency of IS dural injection, Mean(SE)** | | | | | | | | | | | | |
| **50% Face Withdrawl Mechenical Threshold (g) (Ipsilateral)** | | | | | | | | | | | | |
|  | IS  (n=7) | AAV-Cxcr5  +IS  (n=7) | AAV-Cxcr5  +IS+EA  (n=7) | AAV-Cxcr5-NC  +IS+EA  (n=7) | ShRNA-NC  +IS  (n=7) | ShRNA+IS  (n=7) | Effect Size  (95% CI) | P Value | Effect Size  (95% CI) | P Value | Effect Size  (95% CI) | P Value |
| **Day 0 (Baseline)** | 0.37 (0.03) | 0.37 (0.03) | 0.4 (0) | 0.4 (0) | 0.37 (0.03) | 0.4 (0) | NA | >0.9999 | NA | 0.9024 | NA | 0.9024 |
| **Day 1** | 0.33 (0.04) | 0.23 (0.04) | 0.3 (0.05) | 0.37 (0.03) | 0.33 (0.04) | 0.4 (0) | NA | 0.7073 | NA | 0.6521 | NA | 0.9024 |
| **Day 3** | 0.3 (0.05) | 0.07 (0) | 0.3 (0.05) | 0.4 (0) | 0.26 (0.05) | 0.4 (0) | 0.23  (0.03 to 0.42) | 0.0243 | NA | 0.3841 | -0.23  (-0.42 to -0.03) | 0.0243 |
| **Day 5** | 0.19 (0.03) | 0.05 (0.01) | 0.23 (0.04) | 0.37 (0.03) | 0.19 (0.03) | 0.4 (0) | 0.14  (0.01 to 0.27) | 0.0312 | -0.21  (-0.34 to -0.07) | 0.0073 | -0.18  (-0.35 to 0.00) | 0.0499 |
| **Day 7** | 0.22 (0.05) | 0.02 (0) | 0.15 (0.01) | 0.33 (0.04) | 0.15 (0.01) | 0.4 (0) | NA | 0.05 | -0.18  (-0.38 to 0.01) | 0.0641 | -0.13  (-0.18 to -0.08) | 0.0005 |
| **Day 8** | 0.13 (0.02) | 0.02 (0) | 0.18 (0.04) | 0.3 (0.05) | 0.1 (0.02) | 0.37 (0.03) | 0.11  (0.05 to 0.18) | 0.0036 | -0.23  (-0.34 to -0.12) | 0.001 | -0.16  (-0.31 to -0.01) | 0.0403 |
| **50% Face Withdrawl Mechenical Threshold (g) (Contralateral)** | | | | | | | | | | | | |
|  | IS  (n=7) | AAV-Cxcr5  +IS  (n=7) | AAV-Cxcr5  +IS+EA  (n=7) | AAV-Cxcr5-NC  +IS+EA  (n=7) | ShRNA-NC  +IS  (n=7) | ShRNA+IS  (n=7) | Effect Size  (95% CI) | P Value | Effect Size  (95% CI) | P Value | Effect Size  (95% CI) | P Value |
| **Day 0 (Baseline)** | 0.4 (0) | 0.4 (0) | 0.4 (0) | 0.4 (0) | 0.4 (0) | 0.4 (0) | NA | >0.9999 | NA | >0.9999 | NA | >0.9999 |
| **Day 1** | 0.4 (0) | 0.19 (0.03) | 0.33 (0.04) | 0.4 (0) | 0.4 (0) | 0.4 (0) | 0.21  (0.10 to 0.31) | <0.0001 | NA | >0.9999 | -0.14  (-0.24 to -0.03) | 0.0042 |
| **Day 3** | 0.3 (0.05) | 0.07 (0) | 0.33 (0.04) | 0.4 (0) | 0.33 (0.04) | 0.4 (0) | 0.23  (0.12 to 0.33) | <0.0001 | NA | 0.0706 | -0.26  (-0.37 to -0.15) | <0.0001 |
| **Day 5** | 0.19 (0.03) | 0.07 (0) | 0.3 (0.05) | 0.37 (0.03) | 0.26 (0.05) | 0.4 (0) | 0.13  (0.02 to 0.24) | 0.0093 | -0.21  (-0.31 to -0.10) | <0.0001 | -0.23  (-0.34 to -0.12) | <0.0001 |
| **Day 7** | 0.16 (0) | 0.03 (0) | 0.19 (0.03) | 0.37 (0.03) | 0.23 (0.04) | 0.37 (0.03) | 0.13  (0.02 to 0.23) | 0.0119 | -0.21  (-0.31 to -0.10) | <0.0001 | -0.16  (-0.27 to -0.05) | 0.0004 |
| **Day 8** | 0.13 (0.02) | 0.02 (0) | 0.12 (0.02) | 0.37 (0.03) | 0.12 (0.02) | 0.37 (0.03) | 0.11  (0.01 to 0.22) | 0.0305 | -0.23  (-0.34 to -0.12) | <0.0001 | NA | 0.0779 |
| **50% Paw Withdrawl Mechenical Threshold (g) (Ipsilateral)** | | | | | | | | | | | | |
|  | IS  (n=7) | AAV-Cxcr5  +IS  (n=7) | AAV-Cxcr5  +IS+EA  (n=7) | AAV-Cxcr5-NC  +IS+EA  (n=7) | ShRNA-NC  +IS  (n=7) | ShRNA+IS  (n=7) | Effect Size  (95% CI) | P Value | Effect Size  (95% CI) | P Value | Effect Size  (95% CI) | P Value |
| **Day 0 (Baseline)** | 1.69 (0.16) | 1.43 (0.2) | 1.77 (0.15) | 1.74 (0.12) | 1.6 (0.15) | 1.6 (0.15) | NA | 0.5261 | NA | 0.9024 | NA | 0.8162 |
| **Day 1** | 1.31 (0.14) | 1.17 (0.08) | 1.37 (0.13) | 1.46 (0.16) | 1.31 (0.14) | 1.34 (0.18) | NA | 0.9658 | NA | >0.9999 | NA | 0.7372 |
| **Day 3** | 1.06 (0.06) | 0.5 (0.13) | 1.11 (0.07) | 1.31 (0.14) | 0.86 (0.17) | 1.51 (0.14) | 0.55  (0.12 to 0.99) | 0.0167 | -0.46  (-0.91 to -0.01) | 0.0468 | -0.61  (-1.12 to -0.11) | 0.0212 |
| **Day 5** | 0.89 (0.14) | 0.17 (0.04) | 0.8 (0.15) | 1.37 (0.13) | 0.68 (0.17) | 1.26 (0.14) | 0.72  (0.15 to 1.28) | 0.0172 | NA | 0.2512 | -0.63  (-1.24 to -0.03) | 0.0419 |
| **Day 7** | 0.57 (0.11) | 0.07 (0.02) | 0.74 (0.12) | 1.31 (0.14) | 0.44 (0.1) | 1.17 (0.08) | 0.50  (0.05 to 0.95) | 0.0321 | -0.60  (-1.12 to -0.08) | 0.0269 | -0.67  (-1.19 to -0.15) | 0.0162 |
| **Day 8** | 0.49 (0.09) | 0.04 (0) | 0.57 (0.11) | 1.2 (0.14) | 0.22 (0.05) | 1.23 (0.08) | 0.44  (0.10 to 0.79) | 0.0163 | -0.74  (-1.12 to -0.37) | 0.0018 | -0.53  (-0.97 to -0.08) | 0.0237 |
| **50% Paw Withdrawl Mechenical Threshold (g) (Contralateral)** | | | | | | | | | | | | |
|  | IS  (n=7) | AAV-Cxcr5  +IS  (n=7) | AAV-Cxcr5  +IS+EA  (n=7) | AAV-Cxcr5-NC  +IS+EA  (n=7) | ShRNA-NC  +IS  (n=7) | ShRNA+IS  (n=7) | Effect Size  (95% CI) | P Value | Effect Size  (95% CI) | P Value | Effect Size  (95% CI) | P Value |
| **Day 0 (Baseline)** | 1.54 (0.17) | 1.6 (0.15) | 1.83 (0.11) | 1.83 (0.11) | 1.4 (0.17) | 1.77 (0.15) | NA | >0.9999 | NA | 0.9446 | NA | 0.8529 |
| **Day 1** | 1.31 (0.22) | 1.17 (0.08) | 1.34 (0.18) | 1.74 (0.12) | 1.37 (0.13) | 1.6 (0.15) | NA | 0.987 | NA | 0.9099 | NA | 0.9413 |
| **Day 3** | 1.06 (0.18) | 0.3 (0.05) | 1.31 (0.14) | 1.57 (0.11) | 1.26 (0.14) | 1.83 (0.11) | 0.76  (0.07 to 1.45) | 0.0326 | -0.77  (-1.52 to -0.03) | 0.0433 | -1.02  (-1.57 to -0.46) | 0.0027 |
| **Day 5** | 0.94 (0.16) | 0.18 (0.04) | 0.83 (0.11) | 1.37 (0.21) | 1 (0.17) | 1.66 (0.12) | 0.76  (0.19 to 1.33) | 0.0137 | -0.71  (-1.31 to -0.12) | 0.0228 | -0.65  (-1.09 to -0.20) | 0.0088 |
| **Day 7** | 0.77 (0.17) | 0.07 (0) | 0.8 (0.15) | 1.26 (0.14) | 0.79 (0.14) | 1.66 (0.12) | 0.70  (0.02 to 1.38) | 0.0434 | -0.89  (-1.69 to -0.09) | 0.0311 | -0.73  (-1.34 to -0.13) | 0.0212 |
| **Day 8** | 0.57 (0.11) | 0.04 (0) | 0.45 (0.1) | 1.4 (0.17) | 0.54 (0.12) | 1.6 (0.15) | 0.53  (0.09 to 0.97) | 0.0217 | -1.03  (-1.71 to -0.35) | 0.0073 | -0.41  (-0.80 to -0.02) | 0.0389 |
| **Tail Flick Latency (s)** | | | | | | | | | | | | |
|  | IS  (n=7) | AAV-Cxcr5  +IS  (n=7) | AAV-Cxcr5  +IS+EA  (n=7) | AAV-Cxcr5-NC  +IS+EA  (n=7) | ShRNA-NC  +IS  (n=7) | ShRNA+IS  (n=7) | Effect Size  (95% CI) | P Value | Effect Size  (95% CI) | P Value | Effect Size  (95% CI) | P Value |
| **Day 0 (Baseline)** | 3.66 (0.09) | 3.62 (0.12) | 3.56 (0.09) | 3.55 (0.11) | 3.57 (0.06) | 3.5 (0.05) | NA | 0.9997 | NA | 0.6555 | NA | 0.9985 |
| **Day 1** | 2.94 (0.2) | 2.57 (0.2) | 3.12 (0.08) | 3.33 (0.09) | 3.2 (0.06) | 3.41 (0.05) | NA | 0.7829 | NA | 0.3142 | NA | 0.2222 |
| **Day 3** | 2.62 (0.13) | 2.09 (0.27) | 2.86 (0.08) | 3.09 (0.04) | 2.74 (0.08) | 3.19 (0.09) | NA | 0.5211 | -0.57  (-1.10 to -0.05) | 0.0314 | NA | 0.1851 |
| **Day 5** | 2.26 (0.13) | 1.66 (0.1) | 2.21 (0.1) | 2.9 (0.07) | 2.4 (0.11) | 3.03 (0.07) | 0.60  (0.05 to 1.16) | 0.0319 | -0.77  (-1.30 to -0.24) | 0.0049 | -0.55  (-1.03 to -0.07) | 0.0216 |
| **Day 7** | 2.08 (0.12) | 1.51 (0.07) | 2.25 (0.08) | 2.78 (0.11) | 2.11 (0.12) | 2.92 (0.1) | 0.57  (0.09 to 1.05) | 0.0192 | -0.84  (-1.36 to -0.32) | 0.0017 | -0.74  (-1.09 to -0.39) | 0.0001 |
| **Day 8** | 2.45 (0.13) | 1.38 (0.08) | 2.47 (0.16) | 3.06 (0.16) | 2.76 (0.14) | 3.35 (0.06) | 1.19  (0.70 to 1.68) | <0.0001 | -0.78  (-1.24 to -0.32) | 0.002 | -1.09  (-1.73 to -0.45) | 0.0019 |
| **Hot Plate Latency (s)** | | | | | | | | | | | | |
|  | IS  (n=7) | AAV-Cxcr5  +IS  (n=7) | AAV-Cxcr5  +IS+EA  (n=7) | AAV-Cxcr5-NC  +IS+EA  (n=7) | ShRNA-NC  +IS  (n=7) | ShRNA+IS  (n=7) | Effect Size  (95% CI) | P Value | Effect Size  (95% CI) | P Value | Effect Size  (95% CI) | P Value |
| **Day 0 (Baseline)** | 16.77 (0.4) | 17.53 (0.3) | 17.01 (0.93) | 17.05 (1.17) | 16.98 (1.02) | 17.43 (0.72) | NA | 0.7126 | NA | 0.9492 | NA | 0.9889 |
| **Day 1** | 16.3 (1.07) | 15.26 (0.43) | 15.43 (0.97) | 15.82 (1.22) | 15.79 (0.68) | 16.53 (0.48) | NA | 0.8718 | NA | 0.9999 | NA | >0.9999 |
| **Day 3** | 12.48 (0.6) | 8.48 (0.41) | 14.24 (1.07) | 13.6 (1.26) | 13.86 (0.33) | 15.96 (0.35) | 4.00  (0.35 to 7.65) | 0.0338 | -3.48  (-6.93 to -0.02) | 0.0489 | -5.76  (-9.12 to -2.39) | 0.0038 |
| **Day 5** | 10.97 (0.67) | 7.14 (0.48) | 10.48 (0.45) | 14.07 (0.74) | 9.66 (0.54) | 14.73 (0.37) | 3.83  (1.60 to 6.06) | 0.0037 | -3.76  (-7.24 to -0.27) | 0.036 | -3.35  (-5.61 to -1.08) | 0.0081 |
| **Day 7** | 8.97 (0.42) | 6.03 (0.39) | 8.91 (0.58) | 13.33 (0.28) | 9.24 (0.72) | 15.08 (0.4) | 2.94  (1.72 to 4.16) | 0.0006 | -6.11  (-7.34 to -4.87) | <0.0001 | -2.88  (-5.74 to -0.03) | 0.0482 |
| **Day 8** | 7.88 (0.39) | 5.06 (0.42) | 9.92 (0.8) | 13.81 (0.72) | 10.61 (0.83) | 15.08 (0.28) | 2.82  (0.92 to 4.72) | 0.0079 | -7.20  (-9.17 to -5.23) | <0.0001 | -4.86  (-8.52 to -1.21) | 0.0136 |
